# Analysis of Genetic and Environmental Risk Factors and Their Interactions in Korean Patients with Age-Related Macular Degeneration

**DOI:** 10.1371/journal.pone.0132771

**Published:** 2015-07-14

**Authors:** Se Joon Woo, Jeeyun Ahn, Margaux A. Morrison, So Yeon Ahn, Jaebong Lee, Ki Woong Kim, Margaret M. DeAngelis, Kyu Hyung Park

**Affiliations:** 1 Department of Ophthalmology, Seoul National University College of Medicine, Seoul, Korea; 2 Department of Ophthalmology, Seoul National University Bundang Hospital, Seongnam, Korea; 3 Department of Ophthalmology, Seoul Metropolitan Government Seoul National University Boramae Medical Center, Seoul, Korea; 4 Department of Ophthalmology and Visual Sciences, John A. Moran Eye Center, University of Utah, Salt Lake City, Utah, United States of America; 5 Medical Research Collaborating Center, Seoul National University Bundang Hospital, Seongnam, Korea; 6 Department of Psychiatry, Seoul National University College of Medicine, Seoul, Korea; 7 Department of Neuropsychiatry, Seoul National University Bundang Hospital, Seongnam, Korea; University of Florida, UNITED STATES

## Abstract

**Purpose:**

To investigate the association of genetic and environmental factors, and their interactions in Korean patients with exudative age-related macular degeneration (AMD).

**Methods:**

A total of 314 robustly characterized exudative AMD patients, including 111 PCV (polypoidal choroidal vasculopathy) and 154 typical choroidal neovascularization (CNV), and 395 control subjects without any evidence of AMD were enrolled. Full ophthalmologic examinations including fluorescein angiography (FA), indocyanine green angiography (ICG) and optical coherence tomography (OCT) were done, according to which patients were divided into either PCV or typical CNV. Standardized questionnaires were used to collect information regarding underlying systemic diseases, dietary habits, smoking history and body mass index (BMI). A total of 86 SNPs from 31 candidate genes were analyzed. Genotype association and logistic regression analyses were done and stepwise regression models to best predict disease for each AMD subtype were constructed.

**Results:**

Age, spherical equivalent, myopia, and ever smoking were associated with exudative AMD. Age, hypertension, hyperlipidemia, spherical equivalent, and myopia were risk factors for typical CNV, while increased education and ever smoking were significantly associated with PCV (p<.05 for all). Four SNPs, *ARMS2/HTRA1* rs10490924, rs11200638, and rs2736911, and *CFH* rs800292, showed association with exudative AMD. Two of these SNPs, *ARMS2/HTRA1* rs10490924 and rs11200638, showed significant association with typical CNV and PCV specifically. There were no significant interactions between environmental and genetic factors. The most predictive disease model for exudative AMD included age, spherical equivalent, smoking, *CFH* rs800292, and *ARMS2* rs10490924 while that for typical CNV included age, hyperlipidemia, spherical equivalent, and *ARMS2* rs10490924. Smoking, spherical equivalent, and *ARMS2* rs10490924 were the most predictive variables for PCV. When comparing PCV cases to CNV cases, age, BMI, and education were the most predictive risk factors of PCV.

**Conclusions:**

Only one locus, the *ARMS2/HTRA1* was a significant genetic risk factor for Korean exudative AMD, including its subtypes, PCV and typical CNV. Stepwise regression revealed that *CFH* was important to risk of exudative AMD in general but not to any specific subtype. While increased education was a unique risk factor to PCV when compared to CNV, this association was independent of refractive error in this homogenous population from South Korea. No significant interactions between environmental and genetic risk factors were observed.

## Introduction

Age-related macular degeneration (AMD) is characterized by progressive degeneration leading to the loss of retinal pigment epithelial cells and subsequent photoreceptor loss, resulting in irreversible central visual field defect. The development and severity of complex diseases such as AMD is known to be influenced by a number of factors. The high prevalence of AMD in the elderly e.g., those over 60 years of age, indicates that genetic, environmental factors as well as their likely interactions are involved in the pathogenesis.[1,2] Although the largest genome-wide association study (GWAS) meta-analysis and replication to date has confirmed many loci and demonstrated several new loci associated with AMD, the two genetic loci contributing the greatest risk to AMD are complement factor H (*CFH)* (1q32) and *age-related maculopathy susceptibility 2 (ARMS2*)/*Htra serine peptidase 1* (*HTRA1)* (10q26).[2] Among epidemiological factors, the most consistent and strongest reported one is cigarette smoking.[1,3,4] Moreover it has been shown that cigarette smoking interacts with variants in *ARMS2* to amplify the risk of AMD in Caucasians.[5]

Polypoidal choroidal vasculopathy (PCV), which is characterized by inner choroidal vascular network of vessels ending in aneurysmal bulge or outward projection, demonstrates similar clinical manifestations to exudative AMD, but whether it is a subtype of AMD or a distinct disease entity remains controversial.[6,7] PCV is more prevalent in the Asian population with reports showing 40–55% of Japanese exudative AMD, 25% of newly diagnosed Chinese AMD, and 31.7% of Korean exudative AMD patients are PCV.[6,8–10] Numerous studies have examined the correlation between established genetic and environmental risk factors for AMD with PCV, such as *CFH*, *ARMS2/HTRA1* genes, and smoking history, and found significant association.[11–13] A recent meta-analysis also confirmed similarities in genetic risk factors between AMD and PCV, even for the previously inconsistently replicated *CFH* Y402H variant (rs1061170).[14]

Due to the complex disease nature of AMD and PCV, there have been previous reports examining the differential effects of environmental factors, namely smoking, on known genetic risk factors.[5,15,16] However, comprehensive studies focusing on gene-environment interactions for PCV and non-PCV typical choroidal neovascularization (CNV) are scarce in past literature.[15] Hence, this study was performed to investigate the association of genetic and environmental factors and their interaction with Korean exudative AMD patients, especially its subtypes, typical CNV and PCV, to subsequently assess the effect of gene-environment interaction on the pathogenesis of typical CNV and PCV.

## Methods

### Study design

This study was approved by the institutional review board of Seoul National University Bundang Hospital (SNUBH). Written informed consent was obtained from all subjects before participation in the study.

### Patient and control subjects

This study was a case-control study evaluating the genetic and environmental factors of AMD. Exudative AMD patients were recruited from the SNUBH retina clinic from July 2008 to October 2010. All patients underwent comprehensive ophthalmological evaluation, including measurement of best-corrected visual acuity, slit-lamp biomicroscopy, indirect fundus exam, fluorescein angiography (FA), indocyanine green angiography (ICGA, Heidelberg Retina Angiography; Heidelberg Engineering, Heidelberg, Germany), and optical coherence tomography (OCT, Spectralis OCT; Heidelberg Engineering, Heidelberg, Germany). Exudative AMD was diagnosed when there was evidence of choroidal neovascularization associated with nondrusenoid retinal pigment epithelium detachment, serous or hemorrhagic retinal detachment, subretinal hemorrhage, or subretinal exudation.[17] Large geographical atrophy or large drusen without the presence of choroidal neovascularization (CNV) and any secondary CNVs due to myopic degeneration, angioid streak, idiopathic CNV, ocular histoplasmosis syndrome were excluded.

**Fig 1:**
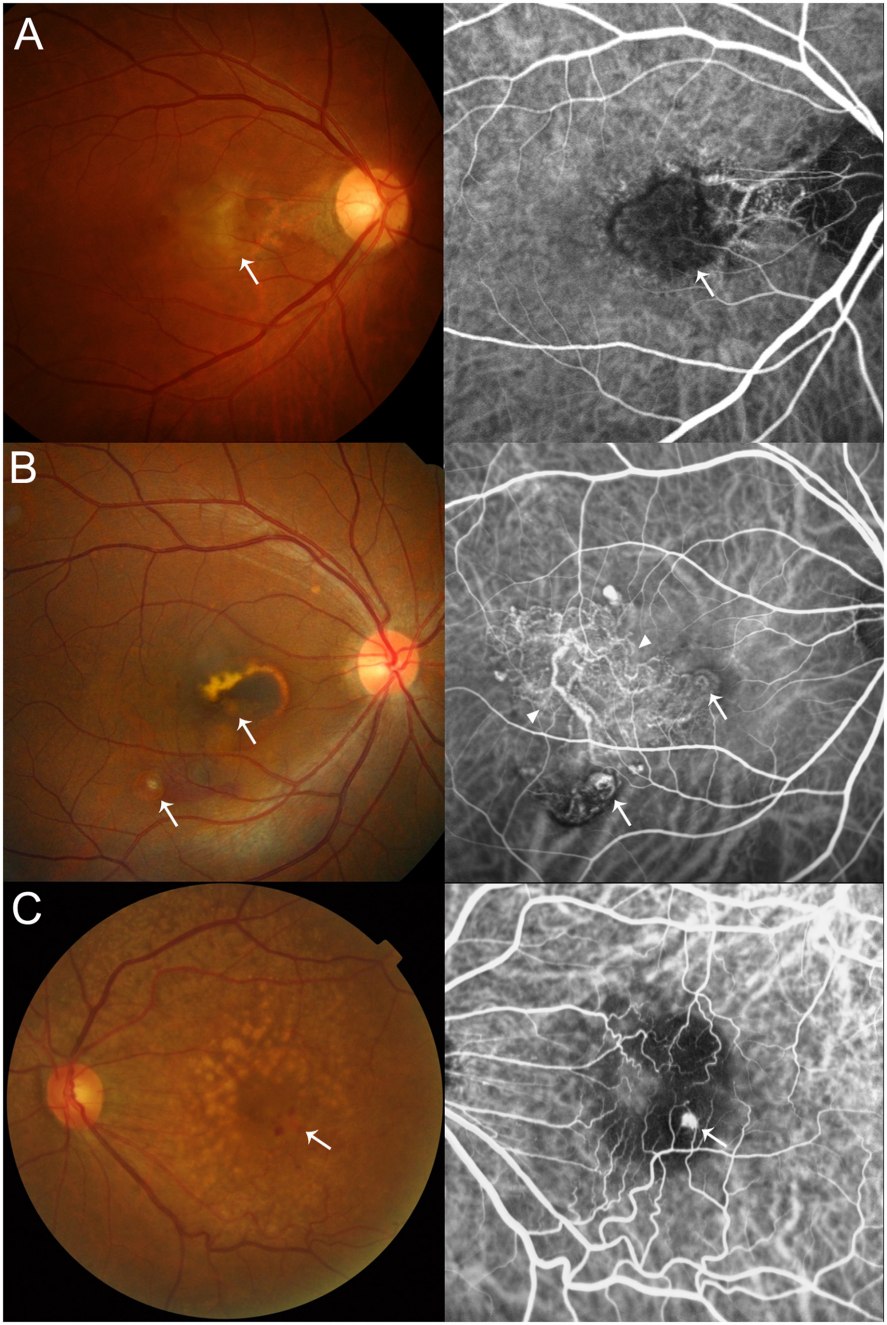
Representative fundus and angiography images of the three age-related macular degeneration subtypes. (A) Typical choroidal neovascularization with surrounding subretinal hemorrhage (white arrow) and evident dye leakage (white arrow) on fluorescein angiography. (B) Polypoidal choroidal vasculopathy showing reddish-orange polyp-like structures (white arrows) and branching choroidal vascular network with polypoidal vascular endings (arrowheads). (C) Retinal angiomatous proliferation with multiple drusen, pinpoint subretinal hemorrhage (white arrow) and chorioretinal anastomosis (white arrow) demonstrated on indocyanine green angiography.

Among the 314 exudative AMD patients recruited, a total of 277 patients underwent ICGA. According to FA, ICGA and OCT findings, this subgroup was divided into three AMD subtypes consisting of typical CNV (n = 157), PCV (n = 112) or retinal angiomatous proliferation (RAP, n = 8) (Fig 1). The typical CNV group was determined by demonstration of choroidal neovascular membrane on FA without any evidence of PCV on ICGA. The PCV group was defined as hyperfluorescent polypoidal choroidal vasculature with branching vascular network on ICGA with concomitant exudation or hemorrhage.[18] The RAP group consisted of cases exhibiting evidence of retinal-retinal or retinal-choroidal anastomosis on ICGA. The 37 patients without ICGA images, RAP patients (n = 8), and typical CNV and PCV patients with missing demographic data (3 patients for typical CNV and 1 patient for PCV) were excluded from the subtype analysis comparing the AMD subtypes, typical CNV and PCV. All diagnoses were made independently by two senior retina specialists (SJW and KYP) and discordant cases were jointly discussed with a third retina specialist (JA).

For the control group, 273 subjects were recruited from people visiting the SNUBH healthcare center for regular medical checkup and 122 subjects were participants of the Korean Longitudinal Study on Health and Aging (KLoSHA); randomly-sampled community-dwelling elderly Koreans aged 65 years or older.[19] Normal control subjects underwent visual acuity examination, fundus photography and/or OCT to ensure that no intermediate sized drusen, RPE changes, retinal diseases, or glaucoma were present. All study subjects were of Korean descent.

### Collection of demographic data

Standardized questionnaires were performed in all patients to collect information regarding age, gender, underlying systemic diseases such as diabetes, hypertension, history of cerebrovascular diseases, and hyperlipidemia, dietary habit, smoking history and body mass index (BMI). Information regarding refractive error was available for a subset of subjects. This included lens status (phakic, pseudophakic, or aphakic), axial length and spherical equivalent. Spherical equivalent (SE, in diopters [D]) was analyzed as the mean of both eyes. When data from only one eye was available, the SE of that eye was used. Myopia was defined as SE ≤ -1 D in at least one eye. Both SE and myopia status were only used when both lenses were phakic. Axial length was analyzed two ways: 1. As the average of both eyes and 2. The right eye (OD) only. According to smoking history, patients were categorized into current, ex- and never smokers. Never smokers were those who had smoked less than 100 cigarettes in the past and ex-smokers had to have quit at least 1 year before the time of examination. An additional sub-analysis categorizing patients into never versus ever smokers was also done.

### Genotype analysis

DNA was extracted from leukocytes in the peripheral blood by DNA extraction kit (QIAamp DNA Maxi kit, Qiagen Inc.). A total of 86 SNPs from 31 candidate genes were analyzed (S1 Table). The SNPs were assayed using multiplex PCR with single base extension primers (iPLEX Gold kit and MassARRAY software, Sequenom, San Diego, CA).

### Statistical analysis

Baseline clinical information and dietary data were compared between patients and controls using the *t*-test, χ^2^ test, or Fisher’s exact test. Genotype data cleaning and analysis was performed using PLINK (http://pngu.mgh.harvard.edu/~purcell/plink/). The SNP data was cleaned by removing SNPs with a low genotyping pass rate (greater than 10% of genotypes missing from the entire cohort) and/or by removing SNPs in those subjects without disease that were not in Hardy Weinberg Equilibrium (HWE p < .0005 (p = 0.05/86 SNPs). Epidemiological variables were tested for their association with disease using logistic regression in SAS (SAS v9.1, Cary, NC: http://www.sas.com/). For the SNP analysis, the minor allele, or less frequent allele, for each SNP was tested for association with disease using the chi square test in PLINK. Correction for multiple testing was performed using two methods: Bonferroni single-step adjusted and FDR BH).[20] Tests for interaction of both genetic and environmental risk factors were performed using interaction terms as well as main effects in the logistic regression model, following the methodology proposed by Keller [21] to control for potential confounders. Specifically, factors that were shown to be significantly associated with each AMD subtype (p < .05) were included in the model along with their corresponding interaction terms. Stepwise logistic regression was performed in order to determine the most significantly associated risk factors. Only those factors shown to be significantly associated with each AMD subtype (p < .05) were included in the stepwise regression model.

## Results

A total of 314 exudative AMD patients, consisting of 157 typical CNV and 112 PCV, and 395 control subjects were recruited. Epidemiological and genetic data were available for all recruited subjects. However, based on presence of cataract, history of intraocular surgery, and recruitment at sub-specialty clinics, lens status (n = 481), spherical equivalent (n = 590), and axial length (n = 385) were only available for subsets of individuals. In the univariate analysis of baseline data, exudative AMD patients were significantly older than control subjects (exudative AMD vs controls = 71.1 vs 68.2 years, P<0.001). (Table 1) Regarding smoking history, exudative AMD showed a positive association (ever smoking: exudative AMD vs controls = 54% vs 42%, P = 0.003). For the subgroup analyses comparing PCV and typical CNV, 154 typical CNV and 111 PCV patients were included. PCV patients were significantly younger than typical CNV patients (PCV vs typical CNV = 67.4 vs 72.6 years, P<0.001) and significantly more likely to have smoking history (ever smoking: PCV vs typical CNV = 64% vs 49%, P = 0.018). There were no significant differences in BMI or the presence of systemic diseases between exudative AMD, control and PCV, typical CNV.

**Table 1:** Distribution of non-genetic variables among studied subjects.

						Exudative AMD				
	Control (N = 395)		Exudative AMD (N = 314)			PCV (N = 111)		Typical CNV (N = 154)		PCV vs. Typical CNV
Variables	N	(%)	N	(%)	P*	N	(%)	N	(%)	P
Age (yr)	68.20±10.13		71.1±8.4		<0.001	67.35±7.34		72.56±8.10		<0.001
Male sex	198	(50)	164	(52)	0.578	61	(55)	86	(56)	0.886
Smoking										
(1) Never	228	(58)	145	(46)		40	(36)	78	(51)	
Ex-	128	(32)	121	(39)		54	(49)	53	(34)	
Current	39	(10)	47	(15)	0.007	17	(15)	23	(15)	0.042
(2) Never	228	(58)	145	(46)		40	(36)	78	(51)	
Ever	167	(42)	168	(54)	0.003	71	(64)	76	(49)	0.018
Education (yr)										
<9	91	(23)	70	(22)		12	(11)	41	(27)	
<12	54	(14)	41	(13)		12	(11)	24	(16)	
<15	122	(31)	83	(27)		32	(29)	38	(25)	
≥15	128	(32)	119	(38)	0.423	55	(50)	51	(33)	0.003
BMI (kg/m2)	23.61±2.96		23.47±3.11		0.542	23.47±2.80		23.66±3.37		0.638
Diabetes	87	(22)	62	(20)	0.462	16	(14)	28	(18)	0.416
Hypertension	182	(46)	164	(52)	0.101	54	(49)	90	(58)	0.114
Cerebrovascular accident	40	(10)	43	(14)	0.199	10	(9)	23	(15)	0.149
Hyperlipidemia	82	(21)	52	(17)	0.134	22	(20)	21	(14)	0.178
Spherical Equivalent (D)°	-0.407±3.13		0.581±1.81		<0.001	0.414±1.72		0.502±1.90		0.737
Myopia°	36	(28)	38	(17)	0.014	16	(18)	31	(21)	1.000
Axial Length (OU)	23.64±1.3		23.60±1.1		0.756	23.57±0.9		23.44±1.1		0.521
Axial Length (OD)	23.66±1.3		23.58±1.1		0.613	23.61±0.9		23.50±1.1		0.310

**Abbreviations**: AMD, age-related macular degeneration; PCV, polypoidal choroidal vasculopathy; CNV, choroidal neovascularization without PCV; BMI, Body Mass Index; D, diopters; OU, oculus uterque (both eyes); OD, oculus dexter (the right eye).

*comparison with controls,

°calculated only in phakic eyes

Of the 86 genotyped SNPs, SNPs *CRP* rs876538, *APP* rs11911934, and *LIPA* rs13500 showed no variation within this cohort and were removed from further analysis. After data cleaning in PLINK, 82 SNPs were tested for association with exudative AMD, typical CNV, PCV, and PCV vs. typical CNV separately among 303 cases and 384 controls. All 82 SNPs were in HWE. After correction for multiple testing, three SNPs in the *ARMS2*/*HTRA1* region (rs10490924, rs11200638, and rs2736911) and *CFH* rs800292 remained significantly associated with exudative AMD (Table 2). For the other analyses, only *ARMS2* rs10490924 and *HTRA1* rs11200638 were significantly associated with typical CNV and PCV after correction for multiple testing (Tables 3 and 4). There were no SNPs significantly associated with PCV compared to typical CNV after correcting for multiple testing. Results of the association analysis for all 82 SNPs are listed in S1 Table.

**Table 2:** Significant Association Results from PLINK comparing Exudative AMD vs. Normal.

CHR	SNP	BP	A1	Freq (A)	Freq (U)	A2	CHISQ	Odds Ratio (95% CI)	UNADJ p	GC p	BONF p	FDR_BH p
1	rs800292	194908856	T	0.290	0.401	C	18.06	0.612 (0.4876–0.7681)	2.14E-05	0.0076	0.0018	0.0006
10	rs2736911	124204345	C	0.898	0.833	T	11.77	1.755 (1.269–2.427)	0.0006	0.0311	0.0493	0.0123
10	rs10490924	124204438	T	0.654	0.396	G	90.05	2.881 (2.309–3.595)	2.32E-21	2.52E-09	1.91E-19	1.91E-19
10	rs11200638	124210534	A	0.655	0.402	G	86.71	2.825 (2.264–3.524)	1.26E-20	4.95E-09	1.03E-18	5.15E-19

SNPs shown above are those that remained significant after correction for multiple testing.

**Abbreviations**: CHR, chromosome; SNP, Single Nucleotide Polymorphism; BP, base pairs; A1, allele 1; Freq (A), frequency of allele 1 in affecteds; Freq (U) frequency of allele 1 in unaffecteds; A2, allele 2; CHISQ, Chi Square value; C.I., confidence interval; GC, Genomic-control; BONF, Bonferroni single-step adjusted; HOLM, Holm (1979) step-down adjusted; SIDAK SS, Sidak single-step adjusted; SIDAK SD, Sidak step-down adjusted; FDR BH, Benjamini & Hochberg (1995) step-up FDR control), and FDR BY (Benjamini & Yekutieli (2001) step-up FDR control).

**Table 3:** Significant Association Results from PLINK comparing CNV vs. Normal.

CHR	SNP	BP	A1	Freq (A)	Freq (U)	A2	CHISQ	Odds Ratio (95% CI)	UNADJ p	GC p	BONF p	FDR_BH p
10	rs10490924	124204438	T	0.637	0.396	G	51.35	2.684(2.04–3.532)	7.73E-13	1.48E-04	6.34E-11	6.34E-11
10	rs11200638	124210534	A	0.637	0.402	G	48.55	2.612 (1.986–3.436)	3.22E-12	2.25E-04	2.64E-10	1.32E-10

SNPs shown above are those that remained significant after correction for multiple testing.

**Abbreviations**: CHR, chromosome; SNP, Single Nucleotide Polymorphism; BP, base pairs; A1, allele 1; Freq (A), frequency of allele 1 in affecteds; Freq (U) frequency of allele 1 in unaffecteds; A2, allele 2; CHISQ, Chi Square value; C.I., confidence interval; GC, Genomic-control; BONF, Bonferroni single-step adjusted; HOLM, Holm (1979) step-down adjusted; SIDAK SS, Sidak single-step adjusted; SIDAK SD, Sidak step-down adjusted; FDR BH, Benjamini & Hochberg (1995) step-up FDR control), and FDR BY (Benjamini & Yekutieli (2001) step-up FDR control).

**Table 4:** Significant Association Results from PLINK comparing PCV vs. Normal.

CHR	SNP	BP	A1	Freq (A)	Freq (U)	A2	CHISQ	Odds Ratio (95% CI)	UNADJ p	GC p	BONF p	FDR_BH p
10	rs10490924	124204438	T	0.637	0.396	G	39.08	2.679 (1.955–3.672)	4.08E-10	2.36E-06	3.34E-08	3.34E-08
10	rs11200638	124210534	A	0.637	0.402	G	36.91	2.607 (1.903–3.572)	1.24E-09	4.50E-06	1.01E-07	5.06E-08

SNPs shown above are those that remained significant after correction for multiple testing.

**Abbreviations**: CHR, chromosome; SNP, Single Nucleotide Polymorphism; BP, base pairs; A1, allele 1; Freq (A), frequency of allele 1 in affecteds; Freq (U) frequency of allele 1 in unaffecteds; A2, allele 2; CHISQ, Chi Square value; C.I., confidence interval; GC, Genomic-control; BONF, Bonferroni single-step adjusted; HOLM, Holm (1979) step-down adjusted; SIDAK SS, Sidak single-step adjusted; SIDAK SD, Sidak step-down adjusted; FDR BH, Benjamini & Hochberg (1995) step-up FDR control), and FDR BY (Benjamini & Yekutieli (2001) step-up FDR control).

As shown in Table 5, logistic regression analysis showed that age and both smoking measures, but not BMI, were significantly associated with increased risk of exudative AMD when compared to normal (p < .05). For phakic eyes, spherical equivalent was associated with increased risk of exudative AMD while myopia showed a protective effect. For typical CNV, significant epidemiological risk factors were age and hypertension, while hyperlipidemia was shown to be protective of typical CNV when compared to normal. For phakic eyes, spherical equivalent was associated with increased risk of CNV, while myopia showed a protective effect. For PCV, increased education and smoking (both measures) were shown to increase risk of disease when compared to normal subjects. Additionally, in phakic eyes, spherical equivalent was associated with increased risk of PCV. When comparing PCV to typical CNV cases, higher education and ever smoking were shown to increase risk of PCV, while increased age was shown to be protective of PCV when compared to typical CNV cases. Interestingly, no significant associations were seen between AMD or its subtypes and axial length by either measure. No significant statistical interaction was seen between any of the risk factors examined, including smoking and the most commonly associated AMD SNPs in *ARMS2*/*HTRA1* and *CFH* after controlling for potential confounders (data not shown).

**Table 5:** Association of non-genetic risk factors using logistic regression.

	Exudative vs. Normal		CNV vs. Normal		PCV vs. Normal		PCV vs. CNV	
Variable	Odds Ratio (95% C.I.)	p value	Odds Ratio (95% C.I.)	p value	Odds Ratio (95% C.I.)	p value	Odds Ratio (95% C.I.)	p value
Age (years)	1.034 (1.017–1.051)	5.25E-05	1.049 (1.028–1.071)	3.76E-06	0.99 (0.969–1.012)	0.3913	0.917 (0.886–0.949)	7.50E-07
BMI (kg/m^2^)	0.985 (0.937–1.035)	0.5420	1.005 (0.946–1.068)	0.8759	0.984 (0.915–1.058)	0.6603	0.981 (0.908–1.061)	0.6378
Cerebrovascular accident	1.317 (0.834–2.079)	0.2376	1.43 (0.826–2.475)	0.2013	0.811 (0.392–1.675)	0.5708	0.567 (0.258–1.244)	0.1571
Diabetes	0.872 (0.605–1.257)	0.4619	0.806 (0.504–1.287)	0.3662	0.588 (0.329–1.051)	0.0730	0.73 (0.375–1.42)	0.3537
Education	1.058 (0.93–1.204)	0.3891	0.939 (0.801–1.101)	0.4355	1.475 (1.199–1.816)	0.0002	1.537 (1.224–1.93)	0.0002
Hypertension	1.282 (0.952–1.726)	0.1015	1.588 (1.092–2.309)	0.0154	1.085 (0.712–1.651)	0.7052	0.683 (0.419–1.112)	0.1254
Hyperlipidemia	0.748 (0.509–1.099)	0.1396	0.584 (0.347–0.983)	0.0429	0.971 (0.578–1.631)	0.9110	1.661 (0.868–3.18)	0.1254
Female sex	0.919 (0.683–1.237)	0.5781	0.809 (0.558–1.172)	0.2624	0.84 (0.552–1.28)	0.4179	1.039 (0.638–1.691)	0.8773
Spherical Equivalent (SE)°	1.187 (1.078–1.307)	0.0005	1.157 (1.031–1.297)	0.0128	1.145 (1.013–1.295)	0.0307	0.974 (0.834–1.136)	0.7347
Myopia°	0.506 (0.301–0.850)	0.0101	0.517 (0.274–0.977)	0.0421	0.553 (0.285–1.073)	0.0799	1.069 (0.510–2.242)	0.8595
Axial Length (Average)	0.970 (0.802–1.174)	0.7551	0.904 (0.701–1.166)	0.4368	1.005 (0.756–1.336)	0.9730	1.142 (0.765–1.706)	0.5167
Axial Length (OD)	0.952 (0.786–1.153)	0.6125	0.860 (0.659–1.121)	0.2644	1.022 (0.773–1.351)	0.8802	1.232 (0.825–1.840)	0.3083
Smoking (2 Level)	1.582 (1.173–2.132)	0.0026	1.297 (0.894–1.882)	0.1707	2.364 (1.533–3.646)	9.89E-05	1.823 (1.11–2.994)	0.0178
Smoking (3 Level)	1.408 (1.136–1.745)	0.0018	1.263 (0.97–1.645)	0.0826	1.718 (1.276–2.313)	0.0004	1.345 (0.956–1.891)	0.0886

PCV = polypoidal choroidal vasculopathy, CNV = choroidal neovascularization without PCV, C.I. = confidence interval, BMI = Body Mass Index

°calculated only in phakic eyes

Additionally, a stepwise regression model was constructed for each AMD association using risk factors determined to be significant for each outcome in single factor analysis (p < .05, Tables 6–9). When comparing exudative AMD cases to controls, the most predictive disease model included age, spherical equivalent as measured in phakic eyes only, smoking (Current/Ex/Never), *CFH* rs8000292, and *ARMS2* rs10490924 (Table 6). The most predictive model for typical CNV included age, hyperlipidemia, spherical equivalent as measured in phakic eyes only, and *ARMS2* rs10490924 (Table 7). The most predictive model of PCV included smoking (Ever/Never), spherical equivalent as measured in phakic eyes only, and *ARMS2* rs10490924 (Table 8). When comparing PCV to CNV cases, age, BMI, and education were the most predictive variables of PCV (Table 9).

**Table 6:** Final stepwise regression model for Exudative AMD vs. Normal.

Risk Factor	Exudative AMD vs. Normal	
	Odds Ratio (95% C.I.)	p value
Age	1.059 (1.027–1.092)	0.0002
Spherical Equivalent°	1.198 (1.068–1.344)	0.0021
Smoking3	1.528 (1.044–2.236)	0.0289
CFH rs800292 (add)	0.567 (0.393–0.818)	0.0024
ARMS2 rs10490924 (add)	2.664 (1.881–3.772)	3.39E-08

Final stepwise regression models were constructed using risk factors that were shown to be significant in single factor analysis (p < .05).

**Abbreviations**: C.I., confidence interval; Smoking3, Current/Ex/Never smoking; add, additive genetic model.

°calculated only in phakic eyes

**Table 7:** Final stepwise regression model for CNV vs. Normal.

Risk Factor	CNV vs. Normal	
	Odds Ratio (95% C.I.)	p value
Age	1.075 (1.038–1.114)	6.82E-05
Hyperlipidemia	0.405 (0.191–0.859)	0.0185
Spherical Equivalent°	1.188 (1.037–1.361)	0.0128
ARMS rs10490924 (add)	2.527 (1.674–3.814)	1.03E-05

Final stepwise regression models were constructed using risk factors that were shown to be significant in single factor analysis (p < .05).

**Abbreviations**: C.I., confidence interval; add, additive genetic model.

°calculated only in phakic eyes

**Table 8:** Final stepwise regression model for PCV vs. Normal.

Risk Factor	PCV vs. Normal	
	Odds Ratio (95% C.I.)	p value
Smoking2	2.460 (1.359–4.453)	0.0030
Spherical Equivalent°	1.187 (1.033–1.364)	0.0156
ARMS2 rs10490924 (add)	2.382 (1.549–3.664)	7.79E-05

Final stepwise regression models were constructed using risk factors that were shown to be significant in single factor analysis (p < .05).

**Abbreviations**: C.I., confidence interval; Smoking2, Ever/Never smoking; add, additive genetic model.

°calculated only in phakic eyes

**Table 9:** Final stepwise regression model for PCV vs. CNV.

Risk Factor	PCV vs. CNV	
	Odds Ratio (95% C.I.)	p value
Age	0.914 (0.880–0.949)	2.65E-06
BMI	0.891 (0.812–0.979)	0.0159
Education	1.457 (1.134–1.872)	0.0032

Final stepwise regression models were constructed using risk factors that were shown to be significant in single factor analysis (p < .05).

**Abbreviations**: C.I., confidence interval; BMI, body mass index.

## Discussion

This is the first study to investigate the association of environmental factors and numerous candidate gene SNPs and their interactions in a relatively large number of Korean AMD patients. Two SNPs, *ARMS2*/*HTRA1* rs10490924 and rs11200638, were significantly associated with exudative AMD and its subtypes, typical CNV and PCV. Age, smoking, hypertension, hyperlipidemia, spherical equivalent (phakic eyes only), and education were significant environmental factors for Korean AMD, while there were no statistically significant interactions between genetic and environmental risk factors affecting risk of AMD.

The *ARMS2*/*HTRA1* genes have been widely reported as the major susceptibility genes for AMD in both Caucasian and Asian ethnicities.[2,22] As for *CFH*, which was the first reported risk gene for Caucasian AMD, there have been controversies surrounding its association with Asian AMD owing to the low frequency of the risk allele in the well-recognized Y402H (rs1061170) variant in Asians.[23–27] Subsequent meta-analyses have shown that this is not the case, with both *ARMS2*/*HTRA1* and *CFH* gene being significantly associated with AMD in Asians, as well as its subtype, PCV.[28,29] In our Korean cohort, the *ARMS2*/*HTRA1* rs10490924 and rs11200638 SNPs were significant risk factors for exudative AMD as well as its subtypes typical CNV and PCV. Like Chen et al., who also studied *CFH* variants in Asians,[28] we found association with *CFH* rs800292 and exudative AMD, and not *CFH* Y402H (rs1061170). These SNPs are independent signals, i.e. not in high LD (r^2^ = 4, data not shown) and show different direction of effect. The lack of significance between *CFH* rs800292 and the specific disease subtypes is likely due to the sample size, while the lack of significance of the *CFH* Y402H variant (rs1061170) in this cohort is likely due to the sample size and the low MAF. The risk allele of Y402H, C, was only found at 7.9% in exudative cases and 9% in controls in this cohort of Koreans, while Caucasians have shown the risk allele to be as frequent as 35%.[30] Meta-analyses increasing sample size to gain adequate statistical power have shown statistically significant differences.[29]

Among environmental factors, age, smoking, hypertension, hyperlipidemia, spherical equivalent, and education were associated with AMD. Most of these factors have already been identified as risk factors for AMD.[31,32] A recent study analyzing data from the Korean National Health and Nutrition Examination survey, a large-scale population-based cross-sectional survey, also reported age and smoking to be associated with late AMD in Koreans.[33] While age was significantly different between our exudative cases and controls, the average age of all subjects was greater than that at which AMD is commonly diagnosed, 50 years, limiting the concern of our controls developing AMD.[34] Age was also included in the stepwise regression models to control for confounding effects. Additionally, of interest, education was found to increase the risk of disease in the PCV subtype when compared to CNV subtypes, which could not be explained by refractive error. Past studies have identified higher education as a protective factor for developing AMD and the reason for the inverse finding in our Korean cohort is difficult to explain as different levels of education may be associated with numerous other factors the association of which may not have been assessed in this study.[17,35,36]

Other notable findings include the protective effect of hyperlipidemia (for typical CNV), diabetes and BMI (for PCV) in single factor analysis. Of these findings, hyperlipidemia and BMI remained important predictors of CNV and PCV, respectively, in stepwise regression. Whether these systemic factors are actually associated with AMD have already been disputed in the past.[37,38] When systemic risk factors for CNV and PCV were compared, CNV was associated with higher prevalence of diabetes compared to PCV.[39,40] The apparent negative association of obesity with AMD was recently reported in Koreans and maybe a distinct feature of Korean AMD.[33] Further larger size population studies will be required to validate our findings.

As for the interaction between smoking and genetic risk factors in AMD, some studies conducted in Caucasians, Japanese and Koreans have advocated the presence of a significant interaction between smoking and AMD risk genes, *CFH* and *ARMS2*/*HTRA1*.[5,15,16] However, most of these studies evaluated smoking-genetic interaction with limited variables in isolation rather than use a regression model with all known genetic and environmental risk factors included. In the study by Nakanishi et al, although logistic regression analysis with the variables age, sex, smoking status, *CFH*, *ARMS2* failed to show a significant interaction between smoking and genetic factors, the authors used the synergy index to suggest a joint smoking-genetic effect.[15] Other studies have failed to find a significant interaction between smoking and genetic risk factors.[41,42] We also found no significant interaction of genetic and environmental risk factors in our Korean cohort when controlling for other significant risk factors.

Although the influence of smoking and high-risk genotypes on the risk of AMD in Koreans should be regarded as significant, the absolute risk of developing AMD and exudative AMD is difficult to estimate accurately in samples collected in a tertiary hospital. Also, our study did not have sufficient statistical power due to the number of patients enrolled and hence well-designed large population-based prospective studies are necessary to reveal the exact interaction of smoking and susceptible genotypes in Koreans. The validity of our result depends on the extent to which our case-control dataset represents the population-based samples of AMD patients and controls. As many clinical parameters such as smoking and education are obtained by interviews, there is a potential for recall bias. Additionally, due to the nature of the recruitment of this study, details of refractive error were not available for the entire cohort examined; therefore, these novel findings should be validated. Future studies should include obtaining refractive error data, specifically spherical equivalent, for study as we have shown it to be an important risk factor in AMD and its subtypes.

In conclusion, variants *ARMS2/HTRA1* rs10490924 and rs11200638 are significant genetic risk factors for Korean exudative AMD and its subtypes CNV and PCV. Age, smoking, hyperlipidemia, spherical equivalent, and education are important environmental factors whilst there was no significant smoking-gene interaction in our Korean cohort.

## Supporting Information

S1 Table Complete SNP Association Results from PLINK. Table a shows the complete association results for all SNPs tested with exudative AMD. Table b shows the complete SNP associations with CNV. Table c shows the complete SNP associations with PCV and Table d shows the complete SNP associations between PCV and CNV.
(DOC)
