# Supplementary material for: Analysis of Genetic and Environmental Risk Factors and Their Interactions in Korean Patients with Age-Related Macular Degeneration
Source: PLoS One. 2015 Jul 14;10(7):e0132771. doi: 10.1371/journal.pone.0132771 (PMC4501798; doi:10.1371/journal.pone.0132771)
Supplement: S1 Table — Table a shows the complete association results for all SNPs tested with exudative AMD. Table b shows the complete SNP associations with CNV. Table c shows the complete SNP associations with PCV and Table d shows the complete SNP associations between PCV and CNV. (DOC) [file pone.0132771.s001.doc]

Supplement Table 1. Association Results from PLINK

1a. Exudative AMD vs. Normal

| **CHR** | **SNP** | **BP** | **A1** | **Freq (A)** | **Freq (U)** | **A2** | **CHISQ** | **Odds Ratio (95% CI)** | **UNADJ p** | **GC p** | **BONF p** | **HOLM p** | **SIDAK_SS p** | **SIDAK_SD p** | **FDR_BH p** | **FDR_BY p** |
| --- | --- | --- | --- | --- | --- | --- | --- | --- | --- | --- | --- | --- | --- | --- | --- | --- |
| 1 | rs1801133 | 11778965 | T | 0.411 | 0.428 | C | 0.43 | 0.9307 (0.75-1.155) | 0.5143 | 0.6821 | 1.0000 | 1.0000 | 1.0000 | 1.0000 | 0.6389 | 1.0000 |
| 1 | rs4845378 | 152811275 | G | 0.856 | 0.833 | T | 1.36 | 1.193 (0.8866-1.606) | 0.2434 | 0.4637 | 1.0000 | 1.0000 | 1.0000 | 1.0000 | 0.5252 | 1.0000 |
| 1 | rs505058 | 154372809 | A | 0.977 | 0.984 | G | 1.01 | 0.673 (0.309-1.466) | 0.3158 | 0.5286 | 1.0000 | 1.0000 | 1.0000 | 1.0000 | 0.5509 | 1.0000 |
| 1 | rs2808635 | 157960833 | G | 0.096 | 0.129 | T | 3.65 | 0.7151 (0.5064-1.01) | 0.0561 | 0.2302 | 1.0000 | 1.0000 | 0.9912 | 0.9627 | 0.1769 | 0.8826 |
| 1 | rs3753394 | 194887540 | T | 0.580 | 0.526 | C | 3.83 | 1.24 (0.9995-1.538) | 0.0504 | 0.2191 | 1.0000 | 1.0000 | 0.9856 | 0.9574 | 0.1755 | 0.8756 |
| 1 | rs7524776 | 194889960 | T | 0.970 | 0.953 | C | 2.68 | 1.611 (0.9055-2.866) | 0.1018 | 0.3041 | 1.0000 | 1.0000 | 0.9999 | 0.9970 | 0.2880 | 1.0000 |
| 1 | rs6680396 | 194899093 | A | 0.753 | 0.669 | G | 11.30 | 1.502 (1.184-1.906) | 0.0008 | 0.0348 | 0.0637 | 0.0606 | 0.0617 | 0.0588 | 0.0127 | 0.0635 |
| 1 | rs800292 | 194908856 | T | 0.290 | 0.401 | C | 18.06 | 0.612 (0.4876-0.7681) | 2.14E-05 | 0.0076 | 0.0018 | 0.0017 | 0.0018 | 0.0017 | 0.0006 | 0.0029 |
| 1 | rs572515 | 194912884 | C | 0.889 | 0.926 | T | 5.32 | 0.6405 (0.4377-0.9373) | 0.0210 | 0.1472 | 1.0000 | 1.0000 | 0.8249 | 0.7643 | 0.1136 | 0.5666 |
| 1 | rs1329423 | 194913010 | G | 0.533 | 0.491 | A | 2.42 | 1.186 (0.9565-1.471) | 0.1198 | 0.3285 | 1.0000 | 1.0000 | 1.0000 | 0.9988 | 0.3275 | 1.0000 |
| 1 | rs3766404 | 194918455 | T | 0.932 | 0.915 | C | 1.36 | 1.273 (0.8481-1.912) | 0.2430 | 0.4633 | 1.0000 | 1.0000 | 1.0000 | 1.0000 | 0.5252 | 1.0000 |
| 1 | rs16840422 | 194919457 | C | 0.929 | 0.915 | T | 0.91 | 1.217 (0.8125-1.822) | 0.3404 | 0.5492 | 1.0000 | 1.0000 | 1.0000 | 1.0000 | 0.5551 | 1.0000 |
| 1 | rs1061147 | 194920947 | C | 0.889 | 0.925 | A | 5.03 | 0.6572 (0.4544-0.9504) | 0.0249 | 0.1590 | 1.0000 | 1.0000 | 0.8738 | 0.8110 | 0.1136 | 0.5666 |
| 1 | rs1061170 | 194925860 | T | 0.891 | 0.932 | C | 6.74 | 0.5997 (0.4063-0.885) | 0.0094 | 0.1029 | 0.7725 | 0.7160 | 0.5398 | 0.5129 | 0.1104 | 0.5507 |
| 1 | rs10922094 | 194928128 | C | 0.891 | 0.921 | G | 3.73 | 0.6953 (0.48-1.007) | 0.0535 | 0.2252 | 1.0000 | 1.0000 | 0.9890 | 0.9588 | 0.1755 | 0.8756 |
| 1 | rs1292471 | 194934051 | A | 0.889 | 0.925 | T | 5.03 | 0.6572 (0.4544-0.9504) | 0.0249 | 0.1590 | 1.0000 | 1.0000 | 0.8738 | 0.8110 | 0.1136 | 0.5666 |
| 1 | rs2860102 | 194934942 | A | 0.889 | 0.923 | T | 4.63 | 0.6695 (0.4636-0.9667) | 0.0314 | 0.1766 | 1.0000 | 1.0000 | 0.9271 | 0.8705 | 0.1304 | 0.6505 |
| 1 | rs2019724 | 194941540 | G | 0.888 | 0.925 | A | 5.47 | 0.6463 (0.4474-0.9336) | 0.0193 | 0.1417 | 1.0000 | 1.0000 | 0.7977 | 0.7493 | 0.1130 | 0.5640 |
| 1 | rs6695321 | 194942484 | G | 0.822 | 0.843 | A | 1.14 | 0.8566 (0.6441-1.139) | 0.2867 | 0.5034 | 1.0000 | 1.0000 | 1.0000 | 1.0000 | 0.5509 | 1.0000 |
| 1 | rs1410997 | 194943786 | T | 0.858 | 0.896 | G | 4.61 | 0.6997 (0.5045-0.9705) | 0.0318 | 0.1775 | 1.0000 | 1.0000 | 0.9293 | 0.8705 | 0.1304 | 0.6505 |
| 1 | rs1831281 | 194947437 | A | 0.305 | 0.358 | G | 4.24 | 0.7878 (0.6276-0.9888) | 0.0395 | 0.1959 | 1.0000 | 1.0000 | 0.9633 | 0.9178 | 0.1543 | 0.7698 |
| 1 | rs2274700 | 194949570 | T | 0.347 | 0.412 | C | 5.77 | 0.7594 (0.6065-0.9508) | 0.0163 | 0.1314 | 1.0000 | 1.0000 | 0.7403 | 0.7038 | 0.1130 | 0.5640 |
| 1 | rs10465586 | 194953952 | A | 0.657 | 0.592 | T | 6.00 | 1.321 (1.057-1.652) | 0.0144 | 0.1241 | 1.0000 | 1.0000 | 0.6942 | 0.6617 | 0.1130 | 0.5640 |
| 1 | rs381974 | 194959295 | C | 0.888 | 0.925 | T | 5.47 | 0.6463 (0.4474-0.9336) | 0.0193 | 0.1417 | 1.0000 | 1.0000 | 0.7977 | 0.7493 | 0.1130 | 0.5640 |
| 1 | rs3753396 | 194962365 | G | 0.513 | 0.487 | A | 0.93 | 1.111 (0.8975-1.374) | 0.3344 | 0.5443 | 1.0000 | 1.0000 | 1.0000 | 1.0000 | 0.5551 | 1.0000 |
| 1 | rs1410996 | 194963556 | C | 0.652 | 0.589 | T | 5.63 | 1.31 (1.048-1.637) | 0.0177 | 0.1363 | 1.0000 | 1.0000 | 0.7688 | 0.7285 | 0.1130 | 0.5640 |
| 1 | rs380390 | 194967674 | G | 0.888 | 0.925 | C | 5.47 | 0.6463 (0.4474-0.9336) | 0.0193 | 0.1417 | 1.0000 | 1.0000 | 0.7977 | 0.7493 | 0.1130 | 0.5640 |
| 1 | rs1329428 | 194969433 | A | 0.375 | 0.438 | G | 5.54 | 0.7701 (0.6194-0.9573) | 0.0186 | 0.1392 | 1.0000 | 1.0000 | 0.7847 | 0.7403 | 0.1130 | 0.5640 |
| 1 | rs424535 | 194975846 | T | 0.421 | 0.437 | A | 0.38 | 0.9347 (0.7536-1.159) | 0.5388 | 0.6994 | 1.0000 | 1.0000 | 1.0000 | 1.0000 | 0.6594 | 1.0000 |
| 1 | rs1065489 | 194976397 | G | 0.490 | 0.520 | T | 1.18 | 0.8887 (0.7181-1.1) | 0.2780 | 0.4957 | 1.0000 | 1.0000 | 1.0000 | 1.0000 | 0.5509 | 1.0000 |
| 1 | rs6428375 | 195139425 | C | 0.817 | 0.817 | T | 0.00 | 1.002 (0.7571-1.326) | 0.9901 | 0.9938 | 1.0000 | 1.0000 | 1.0000 | 1.0000 | 0.9901 | 1.0000 |
| 1 | rs7417769 | 195143081 | G | 0.816 | 0.814 | A | 0.01 | 1.016 (0.7722-1.337) | 0.9087 | 0.9426 | 1.0000 | 1.0000 | 1.0000 | 1.0000 | 0.9314 | 1.0000 |
| 1 | rs1853883 | 195148223 | G | 0.848 | 0.875 | C | 2.06 | 0.7981 (0.5865-1.086) | 0.1510 | 0.3671 | 1.0000 | 1.0000 | 1.0000 | 0.9998 | 0.3869 | 1.0000 |
| 1 | rs1971579 | 195153804 | C | 0.167 | 0.174 | A | 0.13 | 0.9489 (0.7138-1.261) | 0.7179 | 0.8205 | 1.0000 | 1.0000 | 1.0000 | 1.0000 | 0.8409 | 1.0000 |
| 1 | rs4915318 | 195163711 | C | 0.330 | 0.305 | A | 0.96 | 1.121 (0.8919-1.41) | 0.3268 | 0.5380 | 1.0000 | 1.0000 | 1.0000 | 1.0000 | 0.5551 | 1.0000 |
| 1 | rs3790414 | 195186922 | A | 0.334 | 0.316 | T | 0.47 | 1.083 (0.8625-1.36) | 0.4926 | 0.6665 | 1.0000 | 1.0000 | 1.0000 | 1.0000 | 0.6289 | 1.0000 |
| 1 | rs7531555 | 195195933 | T | 0.663 | 0.681 | C | 0.48 | 0.9225 (0.7346-1.158) | 0.4876 | 0.6628 | 1.0000 | 1.0000 | 1.0000 | 1.0000 | 0.6289 | 1.0000 |
| 1 | rs6428379 | 195204159 | C | 0.833 | 0.861 | T | 1.97 | 0.8094 (0.6023-1.088) | 0.1603 | 0.3778 | 1.0000 | 1.0000 | 1.0000 | 0.9998 | 0.3983 | 1.0000 |
| 1 | rs10922152 | 195229629 | T | 0.823 | 0.847 | A | 1.41 | 0.8396 (0.6292-1.12) | 0.2344 | 0.4551 | 1.0000 | 1.0000 | 1.0000 | 1.0000 | 0.5252 | 1.0000 |
| 1 | rs8383 | 225150173 | C | 0.573 | 0.522 | T | 3.47 | 1.226 (0.9892-1.519) | 0.0626 | 0.2422 | 1.0000 | 1.0000 | 0.9950 | 0.9732 | 0.1901 | 0.9487 |
| 3 | rs1049296 | 134977044 | C | 0.723 | 0.724 | T | 0.00 | 0.9948 (0.7838-1.262) | 0.9656 | 0.9784 | 1.0000 | 1.0000 | 1.0000 | 1.0000 | 0.9775 | 1.0000 |
| 4 | rs2071559 | 55687123 | T | 0.718 | 0.711 | C | 0.08 | 1.034 (0.817-1.309) | 0.7792 | 0.8602 | 1.0000 | 1.0000 | 1.0000 | 1.0000 | 0.8445 | 1.0000 |
| 4 | rs13117504 | 110878305 | G | 0.607 | 0.546 | C | 5.16 | 1.285 (1.035-1.596) | 0.0231 | 0.1535 | 1.0000 | 1.0000 | 0.8525 | 0.7907 | 0.1136 | 0.5666 |
| 4 | rs10033900 | 110878516 | C | 0.322 | 0.316 | T | 0.05 | 1.027 (0.8174-1.291) | 0.8172 | 0.8846 | 1.0000 | 1.0000 | 1.0000 | 1.0000 | 0.8702 | 1.0000 |
| 4 | rs11726949 | 110884079 | C | 0.878 | 0.891 | T | 0.54 | 0.8829 (0.6332-1.231) | 0.4624 | 0.6444 | 1.0000 | 1.0000 | 1.0000 | 1.0000 | 0.6289 | 1.0000 |
| 4 | rs3775291 | 187241068 | G | 0.718 | 0.746 | A | 1.40 | 0.8648 (0.6799-1.1) | 0.2365 | 0.4572 | 1.0000 | 1.0000 | 1.0000 | 1.0000 | 0.5252 | 1.0000 |
| 6 | rs9332739 | 32011783 | G | 0.987 | 0.975 | C | 2.34 | 1.896 (0.8243-4.362) | 0.1260 | 0.3365 | 1.0000 | 1.0000 | 1.0000 | 0.9991 | 0.3333 | 1.0000 |
| 6 | rs547154 | 32018917 | C | 0.934 | 0.905 | A | 3.79 | 1.486 (0.9951-2.22) | 0.0517 | 0.2216 | 1.0000 | 1.0000 | 0.9871 | 0.9586 | 0.1755 | 0.8756 |
| 6 | rs4151667 | 32022003 | T | 0.987 | 0.977 | A | 1.91 | 1.794 (0.7747-4.154) | 0.1668 | 0.3851 | 1.0000 | 1.0000 | 1.0000 | 0.9999 | 0.4022 | 1.0000 |
| 6 | rs641153 | 32022159 | C | 0.934 | 0.905 | T | 3.79 | 1.486 (0.9951-2.22) | 0.0517 | 0.2216 | 1.0000 | 1.0000 | 0.9871 | 0.9586 | 0.1755 | 0.8756 |
| 6 | rs699947 | 43844367 | C | 0.742 | 0.746 | A | 0.03 | 0.9773 (0.7656-1.248) | 0.8538 | 0.9079 | 1.0000 | 1.0000 | 1.0000 | 1.0000 | 0.8976 | 1.0000 |
| 6 | rs2010963 | 43846328 | C | 0.426 | 0.434 | G | 0.10 | 0.9658 (0.7782-1.199) | 0.7518 | 0.8425 | 1.0000 | 1.0000 | 1.0000 | 1.0000 | 0.8445 | 1.0000 |
| 6 | rs833069 | 43850557 | G | 0.429 | 0.449 | A | 0.56 | 0.9213 (0.7433-1.142) | 0.4545 | 0.6385 | 1.0000 | 1.0000 | 1.0000 | 1.0000 | 0.6289 | 1.0000 |
| 7 | rs868005 | 73083050 | A | 0.828 | 0.835 | G | 0.09 | 0.9563 (0.7197-1.271) | 0.7583 | 0.8467 | 1.0000 | 1.0000 | 1.0000 | 1.0000 | 0.8445 | 1.0000 |
| 7 | rs884843 | 73083725 | A | 0.629 | 0.620 | G | 0.12 | 1.039 (0.8338-1.295) | 0.7318 | 0.8295 | 1.0000 | 1.0000 | 1.0000 | 1.0000 | 0.8445 | 1.0000 |
| 7 | rs2301995 | 73090076 | C | 0.809 | 0.790 | T | 0.70 | 1.12 (0.8581-1.463) | 0.4033 | 0.5997 | 1.0000 | 1.0000 | 1.0000 | 1.0000 | 0.6241 | 1.0000 |
| 7 | rs13239907 | 73094786 | A | 0.381 | 0.368 | G | 0.25 | 1.057 (0.8484-1.318) | 0.6200 | 0.7555 | 1.0000 | 1.0000 | 1.0000 | 1.0000 | 0.7368 | 1.0000 |
| 7 | rs2856728 | 73108718 | T | 0.718 | 0.725 | C | 0.08 | 0.9671 (0.7626-1.226) | 0.7827 | 0.8625 | 1.0000 | 1.0000 | 1.0000 | 1.0000 | 0.8445 | 1.0000 |
| 10 | rs4146894 | 124145371 | A | 0.737 | 0.655 | G | 10.60 | 1.474 (1.166-1.864) | 0.0011 | 0.0409 | 0.0929 | 0.0873 | 0.0888 | 0.0836 | 0.0155 | 0.0773 |
| 10 | rs1045216 | 124179187 | G | 0.848 | 0.835 | A | 0.46 | 1.107 (0.8263-1.483) | 0.4957 | 0.6687 | 1.0000 | 1.0000 | 1.0000 | 1.0000 | 0.6289 | 1.0000 |
| 10 | rs2736911 | 124204345 | C | 0.898 | 0.833 | T | 11.77 | 1.755 (1.269-2.427) | 0.0006 | 0.0311 | 0.0493 | 0.0475 | 0.0481 | 0.0464 | 0.0123 | 0.0615 |
| 10 | rs10490924 | 124204438 | T | 0.654 | 0.396 | G | 90.05 | 2.881 (2.309-3.595) | 2.32E-21 | 2.52E-09 | 1.91E-19 | 1.91E-19 | INF | INF | 1.91E-19 | 9.51E-19 |
| 10 | rs11200638 | 124210534 | A | 0.655 | 0.402 | G | 86.71 | 2.825 (2.264-3.524) | 1.26E-20 | 4.95E-09 | 1.03E-18 | 1.02E-18 | INF | INF | 5.15E-19 | 2.57E-18 |
| 11 | rs2511989 | 57134901 | A | 0.147 | 0.128 | G | 1.07 | 1.177 (0.864-1.603) | 0.3012 | 0.5161 | 1.0000 | 1.0000 | 1.0000 | 1.0000 | 0.5509 | 1.0000 |
| 11 | rs2511988 | 57135746 | T | 0.743 | 0.759 | C | 0.50 | 0.9154 (0.7157-1.171) | 0.4810 | 0.6580 | 1.0000 | 1.0000 | 1.0000 | 1.0000 | 0.6289 | 1.0000 |
| 11 | rs2373115 | 77768798 | G | 0.629 | 0.608 | T | 0.62 | 1.093 (0.8756-1.364) | 0.4329 | 0.6223 | 1.0000 | 1.0000 | 1.0000 | 1.0000 | 0.6289 | 1.0000 |
| 11 | rs2070045 | 120953300 | T | 0.493 | 0.486 | G | 0.08 | 1.031 (0.8335-1.276) | 0.7762 | 0.8583 | 1.0000 | 1.0000 | 1.0000 | 1.0000 | 0.8445 | 1.0000 |
| 14 | rs1800844 | 72673453 | G | 0.545 | 0.514 | C | 1.25 | 1.13 (0.9124-1.399) | 0.2632 | 0.4822 | 1.0000 | 1.0000 | 1.0000 | 1.0000 | 0.5509 | 1.0000 |
| 14 | rs165932 | 72734606 | C | 0.352 | 0.400 | A | 3.31 | 0.8139 (0.652-1.016) | 0.0687 | 0.2528 | 1.0000 | 1.0000 | 0.9971 | 0.9800 | 0.2012 | 1.0000 |
| 17 | rs9913583 | 1612080 | C | 0.865 | 0.867 | A | 0.02 | 0.9787 (0.7161-1.338) | 0.8925 | 0.9323 | 1.0000 | 1.0000 | 1.0000 | 1.0000 | 0.9264 | 1.0000 |
| 17 | rs1136287 | 1620026 | C | 0.479 | 0.497 | T | 0.48 | 0.9271 (0.7488-1.148) | 0.4873 | 0.6626 | 1.0000 | 1.0000 | 1.0000 | 1.0000 | 0.6289 | 1.0000 |
| 17 | rs2471738 | 41431900 | C | 0.769 | 0.745 | T | 1.07 | 1.141 (0.889-1.464) | 0.3004 | 0.5154 | 1.0000 | 1.0000 | 1.0000 | 1.0000 | 0.5509 | 1.0000 |
| 17 | rs1800764 | 58904261 | T | 0.597 | 0.568 | C | 1.21 | 1.129 (0.9093-1.402) | 0.2719 | 0.4901 | 1.0000 | 1.0000 | 1.0000 | 1.0000 | 0.5509 | 1.0000 |
| 19 | rs429358 | 50103781 | T | 0.924 | 0.909 | C | 0.87 | 1.203 (0.8153-1.776) | 0.3507 | 0.5578 | 1.0000 | 1.0000 | 1.0000 | 1.0000 | 0.5551 | 1.0000 |
| 19 | rs7412 | 50103919 | C | 0.919 | 0.934 | T | 1.03 | 0.8096 (0.5376-1.219) | 0.3113 | 0.5248 | 1.0000 | 1.0000 | 1.0000 | 1.0000 | 0.5509 | 1.0000 |
| 20 | rs1799990 | 4628251 | G | 0.027 | 0.033 | A | 0.31 | 0.8342 (0.4413-1.577) | 0.5766 | 0.7258 | 1.0000 | 1.0000 | 1.0000 | 1.0000 | 0.6953 | 1.0000 |
| 21 | rs466433 | 26465834 | G | 0.198 | 0.184 | A | 0.46 | 1.098 (0.8376-1.439) | 0.4985 | 0.6707 | 1.0000 | 1.0000 | 1.0000 | 1.0000 | 0.6289 | 1.0000 |
| 21 | rs364048 | 26465912 | C | 0.198 | 0.183 | T | 0.51 | 1.104 (0.8419-1.448) | 0.4741 | 0.6530 | 1.0000 | 1.0000 | 1.0000 | 1.0000 | 0.6289 | 1.0000 |
| 21 | rs466448 | 26465979 | G | 0.551 | 0.523 | A | 1.06 | 1.119 (0.9033-1.386) | 0.3039 | 0.5184 | 1.0000 | 1.0000 | 1.0000 | 1.0000 | 0.5509 | 1.0000 |
| 21 | rs364051 | 26466117 | C | 0.213 | 0.198 | T | 0.47 | 1.096 (0.8423-1.426) | 0.4950 | 0.6682 | 1.0000 | 1.0000 | 1.0000 | 1.0000 | 0.6289 | 1.0000 |
| 21 | rs438031 | 26467875 | T | 0.878 | 0.892 | C | 0.63 | 0.8736 (0.6261-1.219) | 0.4267 | 0.6176 | 1.0000 | 1.0000 | 1.0000 | 1.0000 | 0.6289 | 1.0000 |
| 21 | rs463946 | 26468058 | G | 0.201 | 0.182 | C | 0.87 | 1.137 (0.8675-1.49) | 0.3520 | 0.5588 | 1.0000 | 1.0000 | 1.0000 | 1.0000 | 0.5551 | 1.0000 |

**1b. CNV vs. Normal**

| **CHR** | **SNP** | **BP** | **A1** | **Freq (A)** | **Freq (U)** | **A2** | **CHISQ** | **Odds Ratio (95% CI)** | **UNADJ p** | **GC p** | **BONF p** | **HOLM p** | **SIDAK_SS p** | **SIDAK_SD p** | **FDR_BH p** | **FDR_BY p** |
| --- | --- | --- | --- | --- | --- | --- | --- | --- | --- | --- | --- | --- | --- | --- | --- | --- |
| 1 | rs1801133 | 11778965 | T | 0.422 | 0.428 | C | 0.04 | 0.9725 (0.7437-1.272) | 0.8384 | 0.9140 | 1.0000 | 1.0000 | 1.0000 | 1.0000 | 0.9332 | 1.0000 |
| 1 | rs4845378 | 152811275 | G | 0.856 | 0.833 | T | 0.85 | 1.191 (0.8208-1.728) | 0.3572 | 0.6258 | 1.0000 | 1.0000 | 1.0000 | 1.0000 | 0.5526 | 1.0000 |
| 1 | rs505058 | 154372809 | A | 0.980 | 0.984 | G | 0.21 | 0.7958 (0.296-2.14) | 0.6501 | 0.8102 | 1.0000 | 1.0000 | 1.0000 | 1.0000 | 0.8159 | 1.0000 |
| 1 | rs2808635 | 157960833 | G | 0.098 | 0.129 | T | 1.97 | 0.7318 (0.4725-1.133) | 0.1605 | 0.4574 | 1.0000 | 1.0000 | 1.0000 | 0.9997 | 0.3416 | 1.0000 |
| 1 | rs3753394 | 194887540 | T | 0.559 | 0.526 | C | 0.92 | 1.14 (0.8727-1.488) | 0.3369 | 0.6111 | 1.0000 | 1.0000 | 1.0000 | 1.0000 | 0.5417 | 1.0000 |
| 1 | rs7524776 | 194889960 | T | 0.967 | 0.953 | C | 1.09 | 1.46 (0.7152-2.98) | 0.2962 | 0.5801 | 1.0000 | 1.0000 | 1.0000 | 1.0000 | 0.5279 | 1.0000 |
| 1 | rs6680396 | 194899093 | A | 0.758 | 0.669 | G | 8.15 | 1.549 (1.146-2.095) | 0.0043 | 0.1306 | 0.3527 | 0.3398 | 0.2977 | 0.2886 | 0.0882 | 0.4400 |
| 1 | rs800292 | 194908856 | T | 0.301 | 0.401 | C | 9.38 | 0.6428 (0.4839-0.8538) | 0.0022 | 0.1048 | 0.1797 | 0.1753 | 0.1647 | 0.1610 | 0.0599 | 0.2989 |
| 1 | rs572515 | 194912884 | C | 0.881 | 0.926 | T | 5.12 | 0.5917 (0.374-0.9361) | 0.0237 | 0.2309 | 1.0000 | 1.0000 | 0.8598 | 0.8219 | 0.1564 | 0.7806 |
| 1 | rs1329423 | 194913010 | G | 0.533 | 0.491 | A | 1.55 | 1.185 (0.9068-1.548) | 0.2139 | 0.5104 | 1.0000 | 1.0000 | 1.0000 | 0.9999 | 0.4106 | 1.0000 |
| 1 | rs3766404 | 194918455 | T | 0.928 | 0.915 | C | 0.45 | 1.189 (0.7189-1.965) | 0.5003 | 0.7211 | 1.0000 | 1.0000 | 1.0000 | 1.0000 | 0.6647 | 1.0000 |
| 1 | rs16840422 | 194919457 | C | 0.923 | 0.915 | T | 0.19 | 1.115 (0.6793-1.83) | 0.6666 | 0.8196 | 1.0000 | 1.0000 | 1.0000 | 1.0000 | 0.8159 | 1.0000 |
| 1 | rs1061147 | 194920947 | C | 0.882 | 0.925 | A | 4.86 | 0.6127 (0.395-0.9502) | 0.0275 | 0.2430 | 1.0000 | 1.0000 | 0.8980 | 0.8575 | 0.1564 | 0.7806 |
| 1 | rs1061170 | 194925860 | T | 0.880 | 0.932 | C | 7.41 | 0.5371 (0.3414-0.8451) | 0.0065 | 0.1496 | 0.5330 | 0.5070 | 0.4142 | 0.3987 | 0.1066 | 0.5319 |
| 1 | rs10922094 | 194928128 | C | 0.882 | 0.921 | G | 4.03 | 0.6397 (0.4124-0.9924) | 0.0448 | 0.2879 | 1.0000 | 1.0000 | 0.9766 | 0.9491 | 0.2040 | 1.0000 |
| 1 | rs1292471 | 194934051 | A | 0.882 | 0.925 | T | 4.86 | 0.6127 (0.395-0.9502) | 0.0275 | 0.2430 | 1.0000 | 1.0000 | 0.8980 | 0.8575 | 0.1564 | 0.7806 |
| 1 | rs2860102 | 194934942 | A | 0.882 | 0.923 | T | 4.52 | 0.6241 (0.4029-0.9667) | 0.0334 | 0.2601 | 1.0000 | 1.0000 | 0.9385 | 0.8976 | 0.1714 | 0.8553 |
| 1 | rs2019724 | 194941540 | G | 0.879 | 0.925 | A | 5.59 | 0.5939 (0.3842-0.9181) | 0.0180 | 0.2105 | 1.0000 | 1.0000 | 0.7753 | 0.7447 | 0.1479 | 0.7382 |
| 1 | rs6695321 | 194942484 | G | 0.811 | 0.843 | A | 1.71 | 0.7943 (0.562-1.123) | 0.1913 | 0.4890 | 1.0000 | 1.0000 | 1.0000 | 0.9999 | 0.3826 | 1.0000 |
| 1 | rs1410997 | 194943786 | T | 0.856 | 0.896 | G | 3.42 | 0.6891 (0.4637-1.024) | 0.0643 | 0.3273 | 1.0000 | 1.0000 | 0.9957 | 0.9818 | 0.2115 | 1.0000 |
| 1 | rs1831281 | 194947437 | A | 0.304 | 0.358 | G | 2.85 | 0.7827 (0.5888-1.041) | 0.0915 | 0.3715 | 1.0000 | 1.0000 | 0.9996 | 0.9938 | 0.2500 | 1.0000 |
| 1 | rs2274700 | 194949570 | T | 0.353 | 0.412 | C | 3.11 | 0.7775 (0.5876-1.029) | 0.0779 | 0.3505 | 1.0000 | 1.0000 | 0.9987 | 0.9892 | 0.2281 | 1.0000 |
| 1 | rs10465586 | 194953952 | A | 0.650 | 0.592 | T | 3.13 | 1.284 (0.9732-1.695) | 0.0768 | 0.3486 | 1.0000 | 1.0000 | 0.9986 | 0.9892 | 0.2281 | 1.0000 |
| 1 | rs381974 | 194959295 | C | 0.879 | 0.925 | T | 5.59 | 0.5939 (0.3842-0.9181) | 0.0180 | 0.2105 | 1.0000 | 1.0000 | 0.7753 | 0.7447 | 0.1479 | 0.7382 |
| 1 | rs3753396 | 194962365 | G | 0.503 | 0.487 | A | 0.23 | 1.067 (0.8188-1.391) | 0.6298 | 0.7986 | 1.0000 | 1.0000 | 1.0000 | 1.0000 | 0.8070 | 1.0000 |
| 1 | rs1410996 | 194963556 | C | 0.647 | 0.589 | T | 3.00 | 1.28 (0.9677-1.694) | 0.0833 | 0.3590 | 1.0000 | 1.0000 | 0.9992 | 0.9908 | 0.2354 | 1.0000 |
| 1 | rs380390 | 194967674 | G | 0.879 | 0.925 | C | 5.59 | 0.5939 (0.3842-0.9181) | 0.0180 | 0.2105 | 1.0000 | 1.0000 | 0.7753 | 0.7447 | 0.1479 | 0.7382 |
| 1 | rs1329428 | 194969433 | A | 0.376 | 0.438 | G | 3.42 | 0.7741 (0.5899-1.016) | 0.0645 | 0.3276 | 1.0000 | 1.0000 | 0.9958 | 0.9818 | 0.2115 | 1.0000 |
| 1 | rs424535 | 194975846 | T | 0.431 | 0.437 | A | 0.03 | 0.976 (0.7469-1.275) | 0.8588 | 0.9250 | 1.0000 | 1.0000 | 1.0000 | 1.0000 | 0.9332 | 1.0000 |
| 1 | rs1065489 | 194976397 | G | 0.507 | 0.520 | T | 0.15 | 0.9491 (0.728-1.237) | 0.6995 | 0.8380 | 1.0000 | 1.0000 | 1.0000 | 1.0000 | 0.8435 | 1.0000 |
| 1 | rs6428375 | 195139425 | C | 0.815 | 0.817 | T | 0.01 | 0.9849 (0.6935-1.399) | 0.9321 | 0.9640 | 1.0000 | 1.0000 | 1.0000 | 1.0000 | 0.9436 | 1.0000 |
| 1 | rs7417769 | 195143081 | G | 0.811 | 0.814 | A | 0.02 | 0.9783 (0.6972-1.373) | 0.8991 | 0.9464 | 1.0000 | 1.0000 | 1.0000 | 1.0000 | 0.9332 | 1.0000 |
| 1 | rs1853883 | 195148223 | G | 0.827 | 0.875 | C | 4.26 | 0.6819 (0.4733-0.9826) | 0.0391 | 0.2747 | 1.0000 | 1.0000 | 0.9621 | 0.9283 | 0.1888 | 0.9420 |
| 1 | rs1971579 | 195153804 | C | 0.167 | 0.174 | A | 0.08 | 0.9489 (0.6643-1.355) | 0.7730 | 0.8786 | 1.0000 | 1.0000 | 1.0000 | 1.0000 | 0.9144 | 1.0000 |
| 1 | rs4915318 | 195163711 | C | 0.349 | 0.305 | A | 1.95 | 1.222 (0.9219-1.619) | 0.1630 | 0.4600 | 1.0000 | 1.0000 | 1.0000 | 0.9997 | 0.3416 | 1.0000 |
| 1 | rs3790414 | 195186922 | A | 0.363 | 0.316 | T | 2.13 | 1.23 (0.9311-1.624) | 0.1448 | 0.4400 | 1.0000 | 1.0000 | 1.0000 | 0.9996 | 0.3416 | 1.0000 |
| 1 | rs7531555 | 195195933 | T | 0.635 | 0.681 | C | 2.09 | 0.8141 (0.616-1.076) | 0.1479 | 0.4435 | 1.0000 | 1.0000 | 1.0000 | 0.9996 | 0.3416 | 1.0000 |
| 1 | rs6428379 | 195204159 | C | 0.807 | 0.861 | T | 4.79 | 0.6777 (0.4776-0.9615) | 0.0286 | 0.2464 | 1.0000 | 1.0000 | 0.9075 | 0.8611 | 0.1564 | 0.7806 |
| 1 | rs10922152 | 195229629 | T | 0.803 | 0.847 | A | 3.14 | 0.7331 (0.5196-1.034) | 0.0764 | 0.3481 | 1.0000 | 1.0000 | 0.9985 | 0.9892 | 0.2281 | 1.0000 |
| 1 | rs8383 | 225150173 | C | 0.585 | 0.522 | T | 3.47 | 1.29 (0.9865-1.686) | 0.0625 | 0.3240 | 1.0000 | 1.0000 | 0.9950 | 0.9818 | 0.2115 | 1.0000 |
| 3 | rs1049296 | 134977044 | C | 0.693 | 0.724 | T | 1.03 | 0.8605 (0.6439-1.15) | 0.3099 | 0.5908 | 1.0000 | 1.0000 | 1.0000 | 1.0000 | 0.5294 | 1.0000 |
| 4 | rs2071559 | 55687123 | T | 0.709 | 0.711 | C | 0.00 | 0.9914 (0.7404-1.327) | 0.9535 | 0.9754 | 1.0000 | 1.0000 | 1.0000 | 1.0000 | 0.9535 | 1.0000 |
| 4 | rs13117504 | 110878305 | G | 0.621 | 0.546 | C | 4.98 | 1.362 (1.038-1.786) | 0.0256 | 0.2373 | 1.0000 | 1.0000 | 0.8809 | 0.8416 | 0.1564 | 0.7806 |
| 4 | rs10033900 | 110878516 | C | 0.307 | 0.316 | T | 0.08 | 0.9601 (0.7208-1.279) | 0.7806 | 0.8828 | 1.0000 | 1.0000 | 1.0000 | 1.0000 | 0.9144 | 1.0000 |
| 4 | rs11726949 | 110884079 | C | 0.850 | 0.891 | T | 3.45 | 0.6941 (0.4715-1.022) | 0.0633 | 0.3254 | 1.0000 | 1.0000 | 0.9953 | 0.9818 | 0.2115 | 1.0000 |
| 4 | rs3775291 | 187241068 | G | 0.704 | 0.746 | A | 1.98 | 0.8092 (0.6025-1.087) | 0.1592 | 0.4560 | 1.0000 | 1.0000 | 1.0000 | 0.9997 | 0.3416 | 1.0000 |
| 6 | rs9332739 | 32011783 | G | 0.994 | 0.975 | C | 3.78 | 3.856 (0.8927-16.65) | 0.0518 | 0.3031 | 1.0000 | 1.0000 | 0.9872 | 0.9668 | 0.2115 | 1.0000 |
| 6 | rs547154 | 32018917 | C | 0.931 | 0.905 | A | 1.91 | 1.425 (0.8607-2.361) | 0.1666 | 0.4639 | 1.0000 | 1.0000 | 1.0000 | 0.9997 | 0.3416 | 1.0000 |
| 6 | rs4151667 | 32022003 | T | 0.994 | 0.977 | A | 3.42 | 3.648 (0.8413-15.82) | 0.0644 | 0.3274 | 1.0000 | 1.0000 | 0.9957 | 0.9818 | 0.2115 | 1.0000 |
| 6 | rs641153 | 32022159 | C | 0.931 | 0.905 | T | 1.91 | 1.425 (0.8607-2.361) | 0.1666 | 0.4639 | 1.0000 | 1.0000 | 1.0000 | 0.9997 | 0.3416 | 1.0000 |
| 6 | rs699947 | 43844367 | C | 0.709 | 0.746 | A | 1.54 | 0.8298 (0.6175-1.115) | 0.2153 | 0.5117 | 1.0000 | 1.0000 | 1.0000 | 0.9999 | 0.4106 | 1.0000 |
| 6 | rs2010963 | 43846328 | C | 0.395 | 0.434 | G | 1.37 | 0.8504 (0.6484-1.115) | 0.2413 | 0.5349 | 1.0000 | 1.0000 | 1.0000 | 1.0000 | 0.4496 | 1.0000 |
| 6 | rs833069 | 43850557 | G | 0.402 | 0.449 | A | 1.99 | 0.8241 (0.6296-1.079) | 0.1586 | 0.4553 | 1.0000 | 1.0000 | 1.0000 | 0.9997 | 0.3416 | 1.0000 |
| 7 | rs868005 | 73083050 | A | 0.830 | 0.835 | G | 0.03 | 0.9678 (0.6793-1.379) | 0.8561 | 0.9235 | 1.0000 | 1.0000 | 1.0000 | 1.0000 | 0.9332 | 1.0000 |
| 7 | rs884843 | 73083725 | A | 0.625 | 0.620 | G | 0.02 | 1.021 (0.7762-1.343) | 0.8816 | 0.9372 | 1.0000 | 1.0000 | 1.0000 | 1.0000 | 0.9332 | 1.0000 |
| 7 | rs2301995 | 73090076 | C | 0.807 | 0.790 | T | 0.38 | 1.11 (0.796-1.549) | 0.5374 | 0.7440 | 1.0000 | 1.0000 | 1.0000 | 1.0000 | 0.6995 | 1.0000 |
| 7 | rs13239907 | 73094786 | A | 0.395 | 0.368 | G | 0.69 | 1.123 (0.8551-1.474) | 0.4050 | 0.6592 | 1.0000 | 1.0000 | 1.0000 | 1.0000 | 0.5930 | 1.0000 |
| 7 | rs2856728 | 73108718 | T | 0.729 | 0.725 | C | 0.02 | 1.021 (0.7584-1.376) | 0.8889 | 0.9410 | 1.0000 | 1.0000 | 1.0000 | 1.0000 | 0.9332 | 1.0000 |
| 10 | rs4146894 | 124145371 | A | 0.732 | 0.655 | G | 5.94 | 1.439 (1.073-1.93) | 0.0148 | 0.1967 | 1.0000 | 1.0000 | 0.7047 | 0.6819 | 0.1479 | 0.7382 |
| 10 | rs1045216 | 124179187 | G | 0.863 | 0.835 | A | 1.30 | 1.245 (0.8541-1.816) | 0.2535 | 0.5454 | 1.0000 | 1.0000 | 1.0000 | 1.0000 | 0.4619 | 1.0000 |
| 10 | rs2736911 | 124204345 | C | 0.892 | 0.833 | T | 5.94 | 1.655 (1.1-2.488) | 0.0148 | 0.1968 | 1.0000 | 1.0000 | 0.7053 | 0.6819 | 0.1479 | 0.7382 |
| 10 | rs10490924 | 124204438 | T | 0.637 | 0.396 | G | 51.35 | 2.684 (2.04-3.532) | 7.73E-13 | 1.48E-04 | 6.34E-11 | 6.34E-11 | 6.34E-11 | 6.34E-11 | 6.34E-11 | 3.16E-10 |
| 10 | rs11200638 | 124210534 | A | 0.637 | 0.402 | G | 48.55 | 2.612 (1.986-3.436) | 3.22E-12 | 2.25E-04 | 2.64E-10 | 2.60E-10 | 2.64E-10 | 2.60E-10 | 1.32E-10 | 6.58E-10 |
| 11 | rs2511989 | 57134901 | A | 0.167 | 0.128 | G | 2.79 | 1.367 (0.9465-1.975) | 0.0946 | 0.3761 | 1.0000 | 1.0000 | 0.9997 | 0.9943 | 0.2502 | 1.0000 |
| 11 | rs2511988 | 57135746 | T | 0.735 | 0.759 | C | 0.67 | 0.8815 (0.651-1.194) | 0.4143 | 0.6656 | 1.0000 | 1.0000 | 1.0000 | 1.0000 | 0.5961 | 1.0000 |
| 11 | rs2373115 | 77768798 | G | 0.582 | 0.608 | T | 0.62 | 0.8972 (0.6848-1.176) | 0.4314 | 0.6769 | 1.0000 | 1.0000 | 1.0000 | 1.0000 | 0.6099 | 1.0000 |
| 11 | rs2070045 | 120953300 | T | 0.477 | 0.486 | G | 0.06 | 0.9663 (0.7412-1.26) | 0.8001 | 0.8934 | 1.0000 | 1.0000 | 1.0000 | 1.0000 | 0.9241 | 1.0000 |
| 14 | rs1800844 | 72673453 | G | 0.579 | 0.514 | C | 3.65 | 1.298 (0.9931-1.698) | 0.0559 | 0.3114 | 1.0000 | 1.0000 | 0.9911 | 0.9734 | 0.2115 | 1.0000 |
| 14 | rs165932 | 72734606 | C | 0.366 | 0.400 | A | 1.04 | 0.8669 (0.6587-1.141) | 0.3083 | 0.5896 | 1.0000 | 1.0000 | 1.0000 | 1.0000 | 0.5294 | 1.0000 |
| 17 | rs9913583 | 1612080 | C | 0.882 | 0.867 | A | 0.45 | 1.149 (0.7658-1.723) | 0.5026 | 0.7226 | 1.0000 | 1.0000 | 1.0000 | 1.0000 | 0.6647 | 1.0000 |
| 17 | rs1136287 | 1620026 | C | 0.464 | 0.497 | T | 0.97 | 0.875 (0.6708-1.141) | 0.3243 | 0.6017 | 1.0000 | 1.0000 | 1.0000 | 1.0000 | 0.5319 | 1.0000 |
| 17 | rs2471738 | 41431900 | C | 0.791 | 0.745 | T | 2.53 | 1.296 (0.9411-1.784) | 0.1117 | 0.3997 | 1.0000 | 1.0000 | 0.9999 | 0.9976 | 0.2864 | 1.0000 |
| 17 | rs1800764 | 58904261 | T | 0.601 | 0.568 | C | 1.00 | 1.148 (0.8762-1.503) | 0.3171 | 0.5963 | 1.0000 | 1.0000 | 1.0000 | 1.0000 | 0.5306 | 1.0000 |
| 19 | rs429358 | 50103781 | T | 0.918 | 0.909 | C | 0.19 | 1.111 (0.6888-1.792) | 0.6656 | 0.8190 | 1.0000 | 1.0000 | 1.0000 | 1.0000 | 0.8159 | 1.0000 |
| 19 | rs7412 | 50103919 | C | 0.922 | 0.934 | T | 0.48 | 0.8369 (0.5046-1.388) | 0.4899 | 0.7146 | 1.0000 | 1.0000 | 1.0000 | 1.0000 | 0.6647 | 1.0000 |
| 20 | rs1799990 | 4628251 | G | 0.031 | 0.033 | A | 0.03 | 0.9385 (0.4328-2.035) | 0.8724 | 0.9322 | 1.0000 | 1.0000 | 1.0000 | 1.0000 | 0.9332 | 1.0000 |
| 21 | rs466433 | 26465834 | G | 0.206 | 0.184 | A | 0.71 | 1.153 (0.8273-1.607) | 0.4006 | 0.6563 | 1.0000 | 1.0000 | 1.0000 | 1.0000 | 0.5930 | 1.0000 |
| 21 | rs364048 | 26465912 | C | 0.206 | 0.183 | T | 0.76 | 1.159 (0.8316-1.616) | 0.3830 | 0.6441 | 1.0000 | 1.0000 | 1.0000 | 1.0000 | 0.5816 | 1.0000 |
| 21 | rs466448 | 26465979 | G | 0.520 | 0.523 | A | 0.01 | 0.9853 (0.7553-1.285) | 0.9129 | 0.9538 | 1.0000 | 1.0000 | 1.0000 | 1.0000 | 0.9358 | 1.0000 |
| 21 | rs364051 | 26466117 | C | 0.219 | 0.198 | T | 0.60 | 1.136 (0.8218-1.571) | 0.4399 | 0.6825 | 1.0000 | 1.0000 | 1.0000 | 1.0000 | 0.6114 | 1.0000 |
| 21 | rs438031 | 26467875 | T | 0.895 | 0.892 | C | 0.03 | 1.041 (0.676-1.602) | 0.8567 | 0.9238 | 1.0000 | 1.0000 | 1.0000 | 1.0000 | 0.9332 | 1.0000 |
| 21 | rs463946 | 26468058 | G | 0.206 | 0.182 | C | 0.85 | 1.169 (0.8387-1.631) | 0.3558 | 0.6249 | 1.0000 | 1.0000 | 1.0000 | 1.0000 | 0.5526 | 1.0000 |

**1c. PCV vs. Normal**

| **CHR** | **SNP** | **BP** | **A1** | **Freq (A)** | **Freq (U)** | **A2** | **CHISQ** | **Odds Ratio (95% CI)** | **UNADJ p** | **GC p** | **BONF p** | **HOLM p** | **SIDAK_SS p** | **SIDAK_SD p** | **FDR_BH p** | **FDR_BY p** |
| --- | --- | --- | --- | --- | --- | --- | --- | --- | --- | --- | --- | --- | --- | --- | --- | --- |
| 1 | rs1801133 | 11778965 | T | 0.396 | 0.428 | C | 0.70 | 0.8757 (0.6422-1.194) | 0.4012 | 0.5262 | 1.0000 | 1.0000 | 1.0000 | 1.0000 | 0.7443 | 1.0000 |
| 1 | rs4845378 | 152811275 | G | 0.859 | 0.833 | T | 0.78 | 1.213 (0.7885-1.867) | 0.3786 | 0.5062 | 1.0000 | 1.0000 | 1.0000 | 1.0000 | 0.7392 | 1.0000 |
| 1 | rs505058 | 154372809 | A | 0.967 | 0.984 | G | 2.63 | 0.4661 (0.1812-1.199) | 0.1052 | 0.2213 | 1.0000 | 1.0000 | 0.9999 | 0.9995 | 0.5950 | 1.0000 |
| 1 | rs2808635 | 157960833 | G | 0.094 | 0.129 | T | 1.89 | 0.7018 (0.4229-1.165) | 0.1689 | 0.2989 | 1.0000 | 1.0000 | 1.0000 | 1.0000 | 0.5985 | 1.0000 |
| 1 | rs3753394 | 194887540 | T | 0.600 | 0.526 | C | 3.59 | 1.35 (0.9892-1.841) | 0.0581 | 0.1526 | 1.0000 | 1.0000 | 0.9926 | 0.9901 | 0.5689 | 1.0000 |
| 1 | rs7524776 | 194889960 | T | 0.981 | 0.953 | C | 3.35 | 2.564 (0.9024-7.287) | 0.0672 | 0.1671 | 1.0000 | 1.0000 | 0.9967 | 0.9946 | 0.5689 | 1.0000 |
| 1 | rs6680396 | 194899093 | A | 0.741 | 0.669 | G | 3.91 | 1.411 (1.002-1.986) | 0.0480 | 0.1354 | 1.0000 | 1.0000 | 0.9822 | 0.9784 | 0.5689 | 1.0000 |
| 1 | rs800292 | 194908856 | T | 0.293 | 0.401 | C | 8.29 | 0.618 (0.4446-0.859) | 0.0040 | 0.0297 | 0.3262 | 0.3182 | 0.2788 | 0.2730 | 0.1087 | 0.5425 |
| 1 | rs572515 | 194912884 | C | 0.911 | 0.926 | T | 0.49 | 0.8201 (0.4707-1.429) | 0.4834 | 0.5967 | 1.0000 | 1.0000 | 1.0000 | 1.0000 | 0.7563 | 1.0000 |
| 1 | rs1329423 | 194913010 | G | 0.538 | 0.491 | A | 1.48 | 1.21 (0.8899-1.644) | 0.2240 | 0.3586 | 1.0000 | 1.0000 | 1.0000 | 1.0000 | 0.6718 | 1.0000 |
| 1 | rs3766404 | 194918455 | T | 0.929 | 0.915 | C | 0.44 | 1.218 (0.6796-2.182) | 0.5073 | 0.6167 | 1.0000 | 1.0000 | 1.0000 | 1.0000 | 0.7563 | 1.0000 |
| 1 | rs16840422 | 194919457 | C | 0.928 | 0.915 | T | 0.38 | 1.2 (0.6694-2.151) | 0.5400 | 0.6436 | 1.0000 | 1.0000 | 1.0000 | 1.0000 | 0.7665 | 1.0000 |
| 1 | rs1061147 | 194920947 | C | 0.910 | 0.925 | A | 0.46 | 0.8298 (0.4826-1.427) | 0.4993 | 0.6100 | 1.0000 | 1.0000 | 1.0000 | 1.0000 | 0.7563 | 1.0000 |
| 1 | rs1061170 | 194925860 | T | 0.918 | 0.932 | C | 0.49 | 0.8143 (0.4583-1.447) | 0.4830 | 0.5964 | 1.0000 | 1.0000 | 1.0000 | 1.0000 | 0.7563 | 1.0000 |
| 1 | rs10922094 | 194928128 | C | 0.914 | 0.921 | G | 0.11 | 0.9098 (0.5236-1.581) | 0.7373 | 0.8001 | 1.0000 | 1.0000 | 1.0000 | 1.0000 | 0.8754 | 1.0000 |
| 1 | rs1292471 | 194934051 | A | 0.910 | 0.925 | T | 0.46 | 0.8298 (0.4826-1.427) | 0.4993 | 0.6100 | 1.0000 | 1.0000 | 1.0000 | 1.0000 | 0.7563 | 1.0000 |
| 1 | rs2860102 | 194934942 | A | 0.910 | 0.923 | T | 0.37 | 0.8453 (0.4921-1.452) | 0.5422 | 0.6454 | 1.0000 | 1.0000 | 1.0000 | 1.0000 | 0.7665 | 1.0000 |
| 1 | rs2019724 | 194941540 | G | 0.910 | 0.925 | A | 0.46 | 0.8298 (0.4826-1.427) | 0.4993 | 0.6100 | 1.0000 | 1.0000 | 1.0000 | 1.0000 | 0.7563 | 1.0000 |
| 1 | rs6695321 | 194942484 | G | 0.840 | 0.843 | A | 0.02 | 0.9725 (0.642-1.473) | 0.8953 | 0.9209 | 1.0000 | 1.0000 | 1.0000 | 1.0000 | 0.9855 | 1.0000 |
| 1 | rs1410997 | 194943786 | T | 0.868 | 0.896 | G | 1.36 | 0.7605 (0.4793-1.207) | 0.2438 | 0.3789 | 1.0000 | 1.0000 | 1.0000 | 1.0000 | 0.6718 | 1.0000 |
| 1 | rs1831281 | 194947437 | A | 0.307 | 0.358 | G | 1.94 | 0.7927 (0.5715-1.099) | 0.1634 | 0.2927 | 1.0000 | 1.0000 | 1.0000 | 1.0000 | 0.5985 | 1.0000 |
| 1 | rs2274700 | 194949570 | T | 0.343 | 0.412 | C | 3.19 | 0.7453 (0.5396-1.03) | 0.0740 | 0.1773 | 1.0000 | 1.0000 | 0.9982 | 0.9963 | 0.5689 | 1.0000 |
| 1 | rs10465586 | 194953952 | A | 0.660 | 0.592 | T | 3.27 | 1.343 (0.9748-1.849) | 0.0707 | 0.1724 | 1.0000 | 1.0000 | 0.9976 | 0.9956 | 0.5689 | 1.0000 |
| 1 | rs381974 | 194959295 | C | 0.910 | 0.925 | T | 0.46 | 0.8298 (0.4826-1.427) | 0.4993 | 0.6100 | 1.0000 | 1.0000 | 1.0000 | 1.0000 | 0.7563 | 1.0000 |
| 1 | rs3753396 | 194962365 | G | 0.528 | 0.487 | A | 1.14 | 1.18 (0.8701-1.6) | 0.2867 | 0.4212 | 1.0000 | 1.0000 | 1.0000 | 1.0000 | 0.6718 | 1.0000 |
| 1 | rs1410996 | 194963556 | C | 0.654 | 0.589 | T | 2.87 | 1.318 (0.9571-1.815) | 0.0902 | 0.2008 | 1.0000 | 1.0000 | 0.9996 | 0.9987 | 0.5689 | 1.0000 |
| 1 | rs380390 | 194967674 | G | 0.910 | 0.925 | C | 0.46 | 0.8298 (0.4826-1.427) | 0.4993 | 0.6100 | 1.0000 | 1.0000 | 1.0000 | 1.0000 | 0.7563 | 1.0000 |
| 1 | rs1329428 | 194969433 | A | 0.382 | 0.438 | G | 2.09 | 0.795 (0.5822-1.086) | 0.1485 | 0.2753 | 1.0000 | 1.0000 | 1.0000 | 1.0000 | 0.5985 | 1.0000 |
| 1 | rs424535 | 194975846 | T | 0.396 | 0.437 | A | 1.15 | 0.8443 (0.6192-1.151) | 0.2844 | 0.4190 | 1.0000 | 1.0000 | 1.0000 | 1.0000 | 0.6718 | 1.0000 |
| 1 | rs1065489 | 194976397 | G | 0.467 | 0.520 | T | 1.84 | 0.8101 (0.5973-1.099) | 0.1752 | 0.3060 | 1.0000 | 1.0000 | 1.0000 | 1.0000 | 0.5985 | 1.0000 |
| 1 | rs6428375 | 195139425 | C | 0.822 | 0.817 | T | 0.03 | 1.035 (0.6937-1.545) | 0.8649 | 0.8978 | 1.0000 | 1.0000 | 1.0000 | 1.0000 | 0.9716 | 1.0000 |
| 1 | rs7417769 | 195143081 | G | 0.829 | 0.814 | A | 0.24 | 1.106 (0.7396-1.653) | 0.6238 | 0.7112 | 1.0000 | 1.0000 | 1.0000 | 1.0000 | 0.8144 | 1.0000 |
| 1 | rs1853883 | 195148223 | G | 0.873 | 0.875 | C | 0.01 | 0.9788 (0.6198-1.546) | 0.9269 | 0.9448 | 1.0000 | 1.0000 | 1.0000 | 1.0000 | 0.9855 | 1.0000 |
| 1 | rs1971579 | 195153804 | C | 0.160 | 0.174 | A | 0.22 | 0.9062 (0.6004-1.368) | 0.6392 | 0.7234 | 1.0000 | 1.0000 | 1.0000 | 1.0000 | 0.8144 | 1.0000 |
| 1 | rs4915318 | 195163711 | C | 0.302 | 0.305 | A | 0.01 | 0.9868 (0.7087-1.374) | 0.9375 | 0.9528 | 1.0000 | 1.0000 | 1.0000 | 1.0000 | 0.9855 | 1.0000 |
| 1 | rs3790414 | 195186922 | A | 0.305 | 0.316 | T | 0.10 | 0.9471 (0.6803-1.318) | 0.7473 | 0.8078 | 1.0000 | 1.0000 | 1.0000 | 1.0000 | 0.8754 | 1.0000 |
| 1 | rs7531555 | 195195933 | T | 0.691 | 0.681 | C | 0.07 | 1.044 (0.751-1.453) | 0.7960 | 0.8453 | 1.0000 | 1.0000 | 1.0000 | 1.0000 | 0.9193 | 1.0000 |
| 1 | rs6428379 | 195204159 | C | 0.859 | 0.861 | T | 0.01 | 0.982 (0.6344-1.52) | 0.9352 | 0.9511 | 1.0000 | 1.0000 | 1.0000 | 1.0000 | 0.9855 | 1.0000 |
| 1 | rs10922152 | 195229629 | T | 0.846 | 0.847 | A | 0.00 | 0.9915 (0.6482-1.517) | 0.9687 | 0.9764 | 1.0000 | 1.0000 | 1.0000 | 1.0000 | 0.9924 | 1.0000 |
| 1 | rs8383 | 225150173 | C | 0.576 | 0.522 | T | 1.89 | 1.24 (0.9124-1.686) | 0.1688 | 0.2988 | 1.0000 | 1.0000 | 1.0000 | 1.0000 | 0.5985 | 1.0000 |
| 3 | rs1049296 | 134977044 | C | 0.764 | 0.724 | T | 1.38 | 1.236 (0.8672-1.762) | 0.2405 | 0.3755 | 1.0000 | 1.0000 | 1.0000 | 1.0000 | 0.6718 | 1.0000 |
| 4 | rs2071559 | 55687123 | T | 0.731 | 0.711 | C | 0.33 | 1.106 (0.7859-1.555) | 0.5641 | 0.6632 | 1.0000 | 1.0000 | 1.0000 | 1.0000 | 0.7840 | 1.0000 |
| 4 | rs13117504 | 110878305 | G | 0.599 | 0.546 | C | 1.89 | 1.242 (0.9114-1.693) | 0.1694 | 0.2996 | 1.0000 | 1.0000 | 1.0000 | 1.0000 | 0.5985 | 1.0000 |
| 4 | rs10033900 | 110878516 | C | 0.330 | 0.316 | T | 0.16 | 1.067 (0.7718-1.476) | 0.6934 | 0.7659 | 1.0000 | 1.0000 | 1.0000 | 1.0000 | 0.8369 | 1.0000 |
| 4 | rs11726949 | 110884079 | C | 0.906 | 0.891 | T | 0.40 | 1.179 (0.7057-1.97) | 0.5292 | 0.6347 | 1.0000 | 1.0000 | 1.0000 | 1.0000 | 0.7665 | 1.0000 |
| 4 | rs3775291 | 187241068 | G | 0.748 | 0.746 | A | 0.00 | 1.008 (0.7095-1.432) | 0.9641 | 0.9729 | 1.0000 | 1.0000 | 1.0000 | 1.0000 | 0.9924 | 1.0000 |
| 6 | rs9332739 | 32011783 | G | 0.986 | 0.975 | C | 0.85 | 1.767 (0.518-6.03) | 0.3569 | 0.4867 | 1.0000 | 1.0000 | 1.0000 | 1.0000 | 0.7276 | 1.0000 |
| 6 | rs547154 | 32018917 | C | 0.939 | 0.905 | A | 2.36 | 1.608 (0.873-2.961) | 0.1244 | 0.2460 | 1.0000 | 1.0000 | 1.0000 | 0.9999 | 0.5985 | 1.0000 |
| 6 | rs4151667 | 32022003 | T | 0.986 | 0.977 | A | 0.68 | 1.672 (0.4878-5.731) | 0.4085 | 0.5326 | 1.0000 | 1.0000 | 1.0000 | 1.0000 | 0.7443 | 1.0000 |
| 6 | rs641153 | 32022159 | C | 0.939 | 0.905 | T | 2.36 | 1.608 (0.873-2.961) | 0.1244 | 0.2460 | 1.0000 | 1.0000 | 1.0000 | 0.9999 | 0.5985 | 1.0000 |
| 6 | rs699947 | 43844367 | C | 0.786 | 0.746 | A | 1.40 | 1.248 (0.8641-1.802) | 0.2371 | 0.3720 | 1.0000 | 1.0000 | 1.0000 | 1.0000 | 0.6718 | 1.0000 |
| 6 | rs2010963 | 43846328 | C | 0.472 | 0.434 | G | 0.95 | 1.164 (0.8577-1.58) | 0.3291 | 0.4612 | 1.0000 | 1.0000 | 1.0000 | 1.0000 | 0.7101 | 1.0000 |
| 6 | rs833069 | 43850557 | G | 0.467 | 0.449 | A | 0.21 | 1.074 (0.7918-1.457) | 0.6456 | 0.7284 | 1.0000 | 1.0000 | 1.0000 | 1.0000 | 0.8144 | 1.0000 |
| 7 | rs868005 | 73083050 | A | 0.807 | 0.835 | G | 0.92 | 0.8263 (0.5593-1.221) | 0.3377 | 0.4692 | 1.0000 | 1.0000 | 1.0000 | 1.0000 | 0.7101 | 1.0000 |
| 7 | rs884843 | 73083725 | A | 0.613 | 0.620 | G | 0.03 | 0.9712 (0.7105-1.328) | 0.8548 | 0.8901 | 1.0000 | 1.0000 | 1.0000 | 1.0000 | 0.9716 | 1.0000 |
| 7 | rs2301995 | 73090076 | C | 0.811 | 0.790 | T | 0.45 | 1.141 (0.7757-1.677) | 0.5035 | 0.6135 | 1.0000 | 1.0000 | 1.0000 | 1.0000 | 0.7563 | 1.0000 |
| 7 | rs13239907 | 73094786 | A | 0.330 | 0.368 | G | 1.04 | 0.8461 (0.6133-1.167) | 0.3082 | 0.4417 | 1.0000 | 1.0000 | 1.0000 | 1.0000 | 0.7020 | 1.0000 |
| 7 | rs2856728 | 73108718 | T | 0.708 | 0.725 | C | 0.24 | 0.9198 (0.6575-1.287) | 0.6254 | 0.7124 | 1.0000 | 1.0000 | 1.0000 | 1.0000 | 0.8144 | 1.0000 |
| 10 | rs4146894 | 124145371 | A | 0.724 | 0.655 | G | 3.54 | 1.381 (0.9856-1.934) | 0.0601 | 0.1557 | 1.0000 | 1.0000 | 0.9938 | 0.9910 | 0.5689 | 1.0000 |
| 10 | rs1045216 | 124179187 | G | 0.821 | 0.835 | A | 0.23 | 0.9072 (0.6086-1.352) | 0.6326 | 0.7181 | 1.0000 | 1.0000 | 1.0000 | 1.0000 | 0.8144 | 1.0000 |
| 10 | rs2736911 | 124204345 | C | 0.901 | 0.833 | T | 5.89 | 1.819 (1.116-2.966) | 0.0152 | 0.0669 | 1.0000 | 1.0000 | 0.7157 | 0.7023 | 0.3121 | 1.0000 |
| 10 | rs10490924 | 124204438 | T | 0.637 | 0.396 | G | 39.08 | 2.679 (1.955-3.672) | 4.08E-10 | 2.36E-06 | 3.34E-08 | 3.34E-08 | 3.34E-08 | 3.34E-08 | 3.34E-08 | 1.67E-07 |
| 10 | rs11200638 | 124210534 | A | 0.637 | 0.402 | G | 36.91 | 2.607 (1.903-3.572) | 1.24E-09 | 4.50E-06 | 1.01E-07 | 1.00E-07 | 1.01E-07 | 1.00E-07 | 5.06E-08 | 2.53E-07 |
| 11 | rs2511989 | 57134901 | A | 0.127 | 0.128 | G | 0.00 | 0.9978 (0.6324-1.574) | 0.9924 | 0.9943 | 1.0000 | 1.0000 | 1.0000 | 1.0000 | 0.9924 | 1.0000 |
| 11 | rs2511988 | 57135746 | T | 0.741 | 0.759 | C | 0.31 | 0.9058 (0.6391-1.284) | 0.5782 | 0.6747 | 1.0000 | 1.0000 | 1.0000 | 1.0000 | 0.7903 | 1.0000 |
| 11 | rs2373115 | 77768798 | G | 0.675 | 0.608 | T | 3.05 | 1.336 (0.9644-1.85) | 0.0809 | 0.1876 | 1.0000 | 1.0000 | 0.9990 | 0.9977 | 0.5689 | 1.0000 |
| 11 | rs2070045 | 120953300 | T | 0.528 | 0.486 | G | 1.21 | 1.186 (0.8747-1.608) | 0.2718 | 0.4067 | 1.0000 | 1.0000 | 1.0000 | 1.0000 | 0.6718 | 1.0000 |
| 14 | rs1800844 | 72673453 | G | 0.519 | 0.514 | C | 0.01 | 1.018 (0.7512-1.381) | 0.9067 | 0.9295 | 1.0000 | 1.0000 | 1.0000 | 1.0000 | 0.9855 | 1.0000 |
| 14 | rs165932 | 72734606 | C | 0.359 | 0.400 | A | 1.18 | 0.8392 (0.6115-1.152) | 0.2773 | 0.4121 | 1.0000 | 1.0000 | 1.0000 | 1.0000 | 0.6718 | 1.0000 |
| 17 | rs9913583 | 1612080 | C | 0.844 | 0.867 | A | 0.73 | 0.8307 (0.5427-1.272) | 0.3928 | 0.5188 | 1.0000 | 1.0000 | 1.0000 | 1.0000 | 0.7443 | 1.0000 |
| 17 | rs1136287 | 1620026 | C | 0.481 | 0.497 | T | 0.18 | 0.9364 (0.6898-1.271) | 0.6732 | 0.7502 | 1.0000 | 1.0000 | 1.0000 | 1.0000 | 0.8364 | 1.0000 |
| 17 | rs2471738 | 41431900 | C | 0.745 | 0.745 | T | 0.00 | 1.003 (0.7072-1.422) | 0.9878 | 0.9908 | 1.0000 | 1.0000 | 1.0000 | 1.0000 | 0.9924 | 1.0000 |
| 17 | rs1800764 | 58904261 | T | 0.594 | 0.568 | C | 0.48 | 1.115 (0.8183-1.519) | 0.4906 | 0.6028 | 1.0000 | 1.0000 | 1.0000 | 1.0000 | 0.7563 | 1.0000 |
| 19 | rs429358 | 50103781 | T | 0.929 | 0.909 | C | 0.82 | 1.308 (0.7319-2.336) | 0.3638 | 0.4929 | 1.0000 | 1.0000 | 1.0000 | 1.0000 | 0.7276 | 1.0000 |
| 19 | rs7412 | 50103919 | C | 0.901 | 0.934 | T | 2.57 | 0.6478 (0.3797-1.105) | 0.1088 | 0.2261 | 1.0000 | 1.0000 | 0.9999 | 0.9996 | 0.5950 | 1.0000 |
| 20 | rs1799990 | 4628251 | G | 0.019 | 0.033 | A | 0.97 | 0.5885 (0.2025-1.71) | 0.3247 | 0.4571 | 1.0000 | 1.0000 | 1.0000 | 1.0000 | 0.7101 | 1.0000 |
| 21 | rs466433 | 26465834 | G | 0.217 | 0.184 | A | 1.20 | 1.232 (0.8476-1.791) | 0.2734 | 0.4083 | 1.0000 | 1.0000 | 1.0000 | 1.0000 | 0.6718 | 1.0000 |
| 21 | rs364048 | 26465912 | C | 0.217 | 0.183 | T | 1.26 | 1.239 (0.8521-1.802) | 0.2613 | 0.3964 | 1.0000 | 1.0000 | 1.0000 | 1.0000 | 0.6718 | 1.0000 |
| 21 | rs466448 | 26465979 | G | 0.590 | 0.523 | A | 2.93 | 1.308 (0.9612-1.78) | 0.0871 | 0.1964 | 1.0000 | 1.0000 | 0.9994 | 0.9984 | 0.5689 | 1.0000 |
| 21 | rs364051 | 26466117 | C | 0.241 | 0.198 | T | 1.84 | 1.284 (0.8943-1.843) | 0.1749 | 0.3058 | 1.0000 | 1.0000 | 1.0000 | 1.0000 | 0.5985 | 1.0000 |
| 21 | rs438031 | 26467875 | T | 0.882 | 0.892 | C | 0.15 | 0.909 (0.565-1.462) | 0.6940 | 0.7664 | 1.0000 | 1.0000 | 1.0000 | 1.0000 | 0.8369 | 1.0000 |
| 21 | rs463946 | 26468058 | G | 0.222 | 0.182 | C | 1.75 | 1.285 (0.8853-1.865) | 0.1865 | 0.3185 | 1.0000 | 1.0000 | 1.0000 | 1.0000 | 0.6116 | 1.0000 |

**1d. PCV vs. CNV**

| **CHR** | **SNP** | **BP** | **A1** | **Freq (A)** | **Freq (U)** | **A2** | **CHISQ** | **Odds Ratio (95% CI)** | **UNADJ p** | **GC p** | **BONF p** | **HOLM p** | **SIDAK_SS p** | **SIDAK_SD p** | **FDR_BH p** | **FDR_BY p** |
| --- | --- | --- | --- | --- | --- | --- | --- | --- | --- | --- | --- | --- | --- | --- | --- | --- |
| 1 | rs1801133 | 11778965 | T | 0.396 | 0.422 | C | 0.33 | 0.9004 (0.6303-1.286) | 0.5644 | 0.5644 | 1.0000 | 1.0000 | 1.0000 | 1.0000 | 0.9664 | 1.0000 |
| 1 | rs4845378 | 152811275 | G | 0.859 | 0.856 | T | 0.01 | 1.019 (0.6173-1.682) | 0.9418 | 0.9418 | 1.0000 | 1.0000 | 1.0000 | 1.0000 | 0.9717 | 1.0000 |
| 1 | rs505058 | 154372809 | A | 0.967 | 0.980 | G | 0.92 | 0.5857 (0.194-1.768) | 0.3373 | 0.3373 | 1.0000 | 1.0000 | 1.0000 | 1.0000 | 0.8712 | 1.0000 |
| 1 | rs2808635 | 157960833 | G | 0.094 | 0.098 | T | 0.02 | 0.9591 (0.5268-1.746) | 0.8912 | 0.8912 | 1.0000 | 1.0000 | 1.0000 | 1.0000 | 0.9664 | 1.0000 |
| 1 | rs3753394 | 194887540 | T | 0.600 | 0.559 | C | 0.86 | 1.184 (0.829-1.692) | 0.3525 | 0.3525 | 1.0000 | 1.0000 | 1.0000 | 1.0000 | 0.8712 | 1.0000 |
| 1 | rs7524776 | 194889960 | T | 0.981 | 0.967 | C | 0.91 | 1.757 (0.5436-5.677) | 0.3405 | 0.3405 | 1.0000 | 1.0000 | 1.0000 | 1.0000 | 0.8712 | 1.0000 |
| 1 | rs6680396 | 194899093 | A | 0.741 | 0.758 | G | 0.21 | 0.9105 (0.6082-1.363) | 0.6487 | 0.6487 | 1.0000 | 1.0000 | 1.0000 | 1.0000 | 0.9664 | 1.0000 |
| 1 | rs800292 | 194908856 | T | 0.293 | 0.301 | C | 0.04 | 0.9614 (0.655-1.411) | 0.8409 | 0.8409 | 1.0000 | 1.0000 | 1.0000 | 1.0000 | 0.9664 | 1.0000 |
| 1 | rs572515 | 194912884 | C | 0.911 | 0.881 | T | 1.11 | 1.386 (0.754-2.548) | 0.2917 | 0.2917 | 1.0000 | 1.0000 | 1.0000 | 1.0000 | 0.8437 | 1.0000 |
| 1 | rs1329423 | 194913010 | G | 0.538 | 0.533 | A | 0.01 | 1.021 (0.7177-1.453) | 0.9075 | 0.9075 | 1.0000 | 1.0000 | 1.0000 | 1.0000 | 0.9664 | 1.0000 |
| 1 | rs3766404 | 194918455 | T | 0.929 | 0.928 | C | 0.00 | 1.025 (0.5185-2.025) | 0.9443 | 0.9443 | 1.0000 | 1.0000 | 1.0000 | 1.0000 | 0.9717 | 1.0000 |
| 1 | rs16840422 | 194919457 | C | 0.928 | 0.923 | T | 0.05 | 1.076 (0.5474-2.116) | 0.8315 | 0.8315 | 1.0000 | 1.0000 | 1.0000 | 1.0000 | 0.9664 | 1.0000 |
| 1 | rs1061147 | 194920947 | C | 0.910 | 0.882 | A | 1.04 | 1.354 (0.754-2.433) | 0.3087 | 0.3087 | 1.0000 | 1.0000 | 1.0000 | 1.0000 | 0.8437 | 1.0000 |
| 1 | rs1061170 | 194925860 | T | 0.918 | 0.880 | C | 1.83 | 1.516 (0.8268-2.78) | 0.1762 | 0.1762 | 1.0000 | 1.0000 | 1.0000 | 1.0000 | 0.8437 | 1.0000 |
| 1 | rs10922094 | 194928128 | C | 0.914 | 0.882 | G | 1.36 | 1.422 (0.7842-2.579) | 0.2444 | 0.2444 | 1.0000 | 1.0000 | 1.0000 | 1.0000 | 0.8437 | 1.0000 |
| 1 | rs1292471 | 194934051 | A | 0.910 | 0.882 | T | 1.04 | 1.354 (0.754-2.433) | 0.3087 | 0.3087 | 1.0000 | 1.0000 | 1.0000 | 1.0000 | 0.8437 | 1.0000 |
| 1 | rs2860102 | 194934942 | A | 0.910 | 0.882 | T | 1.04 | 1.354 (0.754-2.433) | 0.3087 | 0.3087 | 1.0000 | 1.0000 | 1.0000 | 1.0000 | 0.8437 | 1.0000 |
| 1 | rs2019724 | 194941540 | G | 0.910 | 0.879 | A | 1.27 | 1.397 (0.7797-2.504) | 0.2594 | 0.2594 | 1.0000 | 1.0000 | 1.0000 | 1.0000 | 0.8437 | 1.0000 |
| 1 | rs6695321 | 194942484 | G | 0.840 | 0.811 | A | 0.73 | 1.224 (0.769-1.949) | 0.3931 | 0.3931 | 1.0000 | 1.0000 | 1.0000 | 1.0000 | 0.8712 | 1.0000 |
| 1 | rs1410997 | 194943786 | T | 0.868 | 0.856 | G | 0.14 | 1.104 (0.6627-1.838) | 0.7047 | 0.7047 | 1.0000 | 1.0000 | 1.0000 | 1.0000 | 0.9664 | 1.0000 |
| 1 | rs1831281 | 194947437 | A | 0.307 | 0.304 | G | 0.00 | 1.013 (0.6924-1.481) | 0.9480 | 0.9480 | 1.0000 | 1.0000 | 1.0000 | 1.0000 | 0.9717 | 1.0000 |
| 1 | rs2274700 | 194949570 | T | 0.343 | 0.353 | C | 0.05 | 0.9586 (0.6583-1.396) | 0.8252 | 0.8252 | 1.0000 | 1.0000 | 1.0000 | 1.0000 | 0.9664 | 1.0000 |
| 1 | rs10465586 | 194953952 | A | 0.660 | 0.650 | T | 0.06 | 1.046 (0.723-1.512) | 0.8130 | 0.8130 | 1.0000 | 1.0000 | 1.0000 | 1.0000 | 0.9664 | 1.0000 |
| 1 | rs381974 | 194959295 | C | 0.910 | 0.879 | T | 1.27 | 1.397 (0.7797-2.504) | 0.2594 | 0.2594 | 1.0000 | 1.0000 | 1.0000 | 1.0000 | 0.8437 | 1.0000 |
| 1 | rs3753396 | 194962365 | G | 0.528 | 0.503 | A | 0.31 | 1.105 (0.7785-1.57) | 0.5751 | 0.5751 | 1.0000 | 1.0000 | 1.0000 | 1.0000 | 0.9664 | 1.0000 |
| 1 | rs1410996 | 194963556 | C | 0.654 | 0.647 | T | 0.02 | 1.029 (0.7088-1.495) | 0.8790 | 0.8790 | 1.0000 | 1.0000 | 1.0000 | 1.0000 | 0.9664 | 1.0000 |
| 1 | rs380390 | 194967674 | G | 0.910 | 0.879 | C | 1.27 | 1.397 (0.7797-2.504) | 0.2594 | 0.2594 | 1.0000 | 1.0000 | 1.0000 | 1.0000 | 0.8437 | 1.0000 |
| 1 | rs1329428 | 194969433 | A | 0.382 | 0.376 | G | 0.02 | 1.027 (0.7158-1.473) | 0.8852 | 0.8852 | 1.0000 | 1.0000 | 1.0000 | 1.0000 | 0.9664 | 1.0000 |
| 1 | rs424535 | 194975846 | T | 0.396 | 0.431 | A | 0.64 | 0.8651 (0.6058-1.235) | 0.4250 | 0.4250 | 1.0000 | 1.0000 | 1.0000 | 1.0000 | 0.8937 | 1.0000 |
| 1 | rs1065489 | 194976397 | G | 0.467 | 0.507 | T | 0.78 | 0.8535 (0.601-1.212) | 0.3759 | 0.3759 | 1.0000 | 1.0000 | 1.0000 | 1.0000 | 0.8712 | 1.0000 |
| 1 | rs6428375 | 195139425 | C | 0.822 | 0.815 | T | 0.04 | 1.051 (0.661-1.672) | 0.8327 | 0.8327 | 1.0000 | 1.0000 | 1.0000 | 1.0000 | 0.9664 | 1.0000 |
| 1 | rs7417769 | 195143081 | G | 0.829 | 0.811 | A | 0.27 | 1.13 (0.7144-1.788) | 0.6005 | 0.6005 | 1.0000 | 1.0000 | 1.0000 | 1.0000 | 0.9664 | 1.0000 |
| 1 | rs1853883 | 195148223 | G | 0.873 | 0.827 | C | 2.02 | 1.435 (0.87-2.368) | 0.1557 | 0.1557 | 1.0000 | 1.0000 | 1.0000 | 1.0000 | 0.8437 | 1.0000 |
| 1 | rs1971579 | 195153804 | C | 0.160 | 0.167 | A | 0.04 | 0.9551 (0.5932-1.538) | 0.8499 | 0.8499 | 1.0000 | 1.0000 | 1.0000 | 1.0000 | 0.9664 | 1.0000 |
| 1 | rs4915318 | 195163711 | C | 0.302 | 0.349 | A | 1.24 | 0.8078 (0.5544-1.177) | 0.2658 | 0.2658 | 1.0000 | 1.0000 | 1.0000 | 1.0000 | 0.8437 | 1.0000 |
| 1 | rs3790414 | 195186922 | A | 0.305 | 0.363 | T | 1.87 | 0.7701 (0.5293-1.12) | 0.1717 | 0.1717 | 1.0000 | 1.0000 | 1.0000 | 1.0000 | 0.8437 | 1.0000 |
| 1 | rs7531555 | 195195933 | T | 0.691 | 0.635 | C | 1.71 | 1.283 (0.8824-1.865) | 0.1915 | 0.1915 | 1.0000 | 1.0000 | 1.0000 | 1.0000 | 0.8437 | 1.0000 |
| 1 | rs6428379 | 195204159 | C | 0.859 | 0.807 | T | 2.32 | 1.449 (0.8972-2.34) | 0.1280 | 0.1280 | 1.0000 | 1.0000 | 1.0000 | 1.0000 | 0.8437 | 1.0000 |
| 1 | rs10922152 | 195229629 | T | 0.846 | 0.803 | A | 1.59 | 1.352 (0.8446-2.166) | 0.2077 | 0.2077 | 1.0000 | 1.0000 | 1.0000 | 1.0000 | 0.8437 | 1.0000 |
| 1 | rs8383 | 225150173 | C | 0.576 | 0.585 | T | 0.05 | 0.9618 (0.6745-1.371) | 0.8295 | 0.8295 | 1.0000 | 1.0000 | 1.0000 | 1.0000 | 0.9664 | 1.0000 |
| 3 | rs1049296 | 134977044 | C | 0.764 | 0.693 | T | 3.18 | 1.437 (0.9636-2.142) | 0.0748 | 0.0748 | 1.0000 | 1.0000 | 0.9983 | 0.9978 | 0.8437 | 1.0000 |
| 4 | rs2071559 | 55687123 | T | 0.731 | 0.709 | C | 0.30 | 1.115 (0.7542-1.649) | 0.5845 | 0.5845 | 1.0000 | 1.0000 | 1.0000 | 1.0000 | 0.9664 | 1.0000 |
| 4 | rs13117504 | 110878305 | G | 0.599 | 0.621 | C | 0.25 | 0.9122 (0.6371-1.306) | 0.6157 | 0.6157 | 1.0000 | 1.0000 | 1.0000 | 1.0000 | 0.9664 | 1.0000 |
| 4 | rs10033900 | 110878516 | C | 0.330 | 0.307 | T | 0.31 | 1.112 (0.7638-1.618) | 0.5800 | 0.5800 | 1.0000 | 1.0000 | 1.0000 | 1.0000 | 0.9664 | 1.0000 |
| 4 | rs11726949 | 110884079 | C | 0.906 | 0.850 | T | 3.53 | 1.698 (0.973-2.965) | 0.0602 | 0.0602 | 1.0000 | 1.0000 | 0.9939 | 0.9931 | 0.8437 | 1.0000 |
| 4 | rs3775291 | 187241068 | G | 0.748 | 0.704 | A | 1.18 | 1.246 (0.8376-1.853) | 0.2774 | 0.2774 | 1.0000 | 1.0000 | 1.0000 | 1.0000 | 0.8437 | 1.0000 |
| 6 | rs9332739 | 32011783 | G | 0.986 | 0.994 | C | 0.76 | 0.4583 (0.07593-2.767) | 0.3834 | 0.3834 | 1.0000 | 1.0000 | 1.0000 | 1.0000 | 0.8712 | 1.0000 |
| 6 | rs547154 | 32018917 | C | 0.939 | 0.931 | A | 0.11 | 1.128 (0.5518-2.306) | 0.7413 | 0.7413 | 1.0000 | 1.0000 | 1.0000 | 1.0000 | 0.9664 | 1.0000 |
| 6 | rs4151667 | 32022003 | T | 0.986 | 0.994 | A | 0.76 | 0.4583 (0.07593-2.767) | 0.3834 | 0.3834 | 1.0000 | 1.0000 | 1.0000 | 1.0000 | 0.8712 | 1.0000 |
| 6 | rs641153 | 32022159 | C | 0.939 | 0.931 | T | 0.11 | 1.128 (0.5518-2.306) | 0.7413 | 0.7413 | 1.0000 | 1.0000 | 1.0000 | 1.0000 | 0.9664 | 1.0000 |
| 6 | rs699947 | 43844367 | C | 0.786 | 0.709 | A | 3.80 | 1.504 (0.9963-2.27) | 0.0513 | 0.0513 | 1.0000 | 1.0000 | 0.9867 | 0.9860 | 0.8437 | 1.0000 |
| 6 | rs2010963 | 43846328 | C | 0.472 | 0.395 | G | 3.03 | 1.369 (0.9605-1.951) | 0.0820 | 0.0820 | 1.0000 | 1.0000 | 0.9991 | 0.9987 | 0.8437 | 1.0000 |
| 6 | rs833069 | 43850557 | G | 0.467 | 0.402 | A | 2.16 | 1.303 (0.9153-1.856) | 0.1415 | 0.1415 | 1.0000 | 1.0000 | 1.0000 | 1.0000 | 0.8437 | 1.0000 |
| 7 | rs868005 | 73083050 | A | 0.807 | 0.830 | G | 0.47 | 0.8539 (0.5428-1.343) | 0.4939 | 0.4939 | 1.0000 | 1.0000 | 1.0000 | 1.0000 | 0.9664 | 1.0000 |
| 7 | rs884843 | 73083725 | A | 0.613 | 0.625 | G | 0.07 | 0.9512 (0.663-1.365) | 0.7860 | 0.7860 | 1.0000 | 1.0000 | 1.0000 | 1.0000 | 0.9664 | 1.0000 |
| 7 | rs2301995 | 73090076 | C | 0.811 | 0.807 | T | 0.01 | 1.027 (0.6575-1.605) | 0.9064 | 0.9064 | 1.0000 | 1.0000 | 1.0000 | 1.0000 | 0.9664 | 1.0000 |
| 7 | rs13239907 | 73094786 | A | 0.330 | 0.395 | G | 2.29 | 0.7537 (0.5223-1.088) | 0.1302 | 0.1302 | 1.0000 | 1.0000 | 1.0000 | 1.0000 | 0.8437 | 1.0000 |
| 7 | rs2856728 | 73108718 | T | 0.708 | 0.729 | C | 0.28 | 0.9005 (0.6105-1.328) | 0.5970 | 0.5970 | 1.0000 | 1.0000 | 1.0000 | 1.0000 | 0.9664 | 1.0000 |
| 10 | rs4146894 | 124145371 | A | 0.724 | 0.732 | G | 0.04 | 0.9594 (0.6467-1.423) | 0.8366 | 0.8366 | 1.0000 | 1.0000 | 1.0000 | 1.0000 | 0.9664 | 1.0000 |
| 10 | rs1045216 | 124179187 | G | 0.821 | 0.863 | A | 1.69 | 0.7285 (0.4513-1.176) | 0.1935 | 0.1935 | 1.0000 | 1.0000 | 1.0000 | 1.0000 | 0.8437 | 1.0000 |
| 10 | rs2736911 | 124204345 | C | 0.901 | 0.892 | T | 0.10 | 1.099 (0.6171-1.959) | 0.7476 | 0.7476 | 1.0000 | 1.0000 | 1.0000 | 1.0000 | 0.9664 | 1.0000 |
| 10 | rs10490924 | 124204438 | T | 0.637 | 0.637 | G | 0.00 | 0.998 (0.6934-1.437) | 0.9914 | 0.9914 | 1.0000 | 1.0000 | 1.0000 | 1.0000 | 0.9914 | 1.0000 |
| 10 | rs11200638 | 124210534 | A | 0.637 | 0.637 | G | 0.00 | 0.998 (0.6934-1.437) | 0.9914 | 0.9914 | 1.0000 | 1.0000 | 1.0000 | 1.0000 | 0.9914 | 1.0000 |
| 11 | rs2511989 | 57134901 | A | 0.127 | 0.167 | G | 1.51 | 0.7297 (0.4411-1.207) | 0.2187 | 0.2187 | 1.0000 | 1.0000 | 1.0000 | 1.0000 | 0.8437 | 1.0000 |
| 11 | rs2511988 | 57135746 | T | 0.741 | 0.735 | C | 0.02 | 1.028 (0.6899-1.531) | 0.8933 | 0.8933 | 1.0000 | 1.0000 | 1.0000 | 1.0000 | 0.9664 | 1.0000 |
| 11 | rs2373115 | 77768798 | G | 0.675 | 0.582 | T | 4.46 | 1.489 (1.028-2.155) | 0.0347 | 0.0347 | 1.0000 | 1.0000 | 0.9448 | 0.9448 | 0.8437 | 1.0000 |
| 11 | rs2070045 | 120953300 | T | 0.528 | 0.477 | G | 1.31 | 1.227 (0.8643-1.743) | 0.2520 | 0.2520 | 1.0000 | 1.0000 | 1.0000 | 1.0000 | 0.8437 | 1.0000 |
| 14 | rs1800844 | 72673453 | G | 0.519 | 0.579 | C | 1.83 | 0.7843 (0.5512-1.116) | 0.1767 | 0.1767 | 1.0000 | 1.0000 | 1.0000 | 1.0000 | 0.8437 | 1.0000 |
| 14 | rs165932 | 72734606 | C | 0.359 | 0.366 | A | 0.03 | 0.968 (0.6723-1.394) | 0.8610 | 0.8610 | 1.0000 | 1.0000 | 1.0000 | 1.0000 | 0.9664 | 1.0000 |
| 17 | rs9913583 | 1612080 | C | 0.844 | 0.882 | A | 1.57 | 0.7232 (0.4349-1.203) | 0.2106 | 0.2106 | 1.0000 | 1.0000 | 1.0000 | 1.0000 | 0.8437 | 1.0000 |
| 17 | rs1136287 | 1620026 | C | 0.481 | 0.464 | T | 0.14 | 1.07 (0.7528-1.521) | 0.7056 | 0.7056 | 1.0000 | 1.0000 | 1.0000 | 1.0000 | 0.9664 | 1.0000 |
| 17 | rs2471738 | 41431900 | C | 0.745 | 0.791 | T | 1.48 | 0.7738 (0.5115-1.171) | 0.2241 | 0.2241 | 1.0000 | 1.0000 | 1.0000 | 1.0000 | 0.8437 | 1.0000 |
| 17 | rs1800764 | 58904261 | T | 0.594 | 0.601 | C | 0.03 | 0.9714 (0.6797-1.388) | 0.8736 | 0.8736 | 1.0000 | 1.0000 | 1.0000 | 1.0000 | 0.9664 | 1.0000 |
| 19 | rs429358 | 50103781 | T | 0.929 | 0.918 | C | 0.23 | 1.177 (0.6048-2.29) | 0.6313 | 0.6313 | 1.0000 | 1.0000 | 1.0000 | 1.0000 | 0.9664 | 1.0000 |
| 19 | rs7412 | 50103919 | C | 0.901 | 0.922 | T | 0.67 | 0.7741 (0.419-1.43) | 0.4125 | 0.4125 | 1.0000 | 1.0000 | 1.0000 | 1.0000 | 0.8901 | 1.0000 |
| 20 | rs1799990 | 4628251 | G | 0.019 | 0.031 | A | 0.60 | 0.6271 (0.1905-2.064) | 0.4388 | 0.4388 | 1.0000 | 1.0000 | 1.0000 | 1.0000 | 0.8995 | 1.0000 |
| 21 | rs466433 | 26465834 | G | 0.217 | 0.206 | A | 0.09 | 1.069 (0.6965-1.64) | 0.7606 | 0.7606 | 1.0000 | 1.0000 | 1.0000 | 1.0000 | 0.9664 | 1.0000 |
| 21 | rs364048 | 26465912 | C | 0.217 | 0.206 | T | 0.09 | 1.069 (0.6965-1.64) | 0.7606 | 0.7606 | 1.0000 | 1.0000 | 1.0000 | 1.0000 | 0.9664 | 1.0000 |
| 21 | rs466448 | 26465979 | G | 0.590 | 0.520 | A | 2.46 | 1.328 (0.9316-1.892) | 0.1165 | 0.1165 | 1.0000 | 1.0000 | 1.0000 | 0.9999 | 0.8437 | 1.0000 |
| 21 | rs364051 | 26466117 | C | 0.241 | 0.219 | T | 0.33 | 1.13 (0.7458-1.712) | 0.5642 | 0.5642 | 1.0000 | 1.0000 | 1.0000 | 1.0000 | 0.9664 | 1.0000 |
| 21 | rs438031 | 26467875 | T | 0.882 | 0.895 | C | 0.23 | 0.8736 (0.5014-1.522) | 0.6331 | 0.6331 | 1.0000 | 1.0000 | 1.0000 | 1.0000 | 0.9664 | 1.0000 |
| 21 | rs463946 | 26468058 | G | 0.222 | 0.206 | C | 0.19 | 1.099 (0.7173-1.683) | 0.6652 | 0.6652 | 1.0000 | 1.0000 | 1.0000 | 1.0000 | 0.9664 | 1.0000 |

**Abbreviations:** CHR, chromosome; SNP, Single Nucleotide Polymorphism; BP, base pairs; A1, allele 1; Freq (A), frequency of allele 1 in affecteds; Freq (U) frequency of allele 1 in unaffecteds; A2, allele 2; CHISQ, Chi Square value; C.I., confidence interval; GC, Genomic-control; BONF, Bonferroni single-step adjusted; HOLM, Holm (1979) step-down adjusted; SIDAK SS, Sidak single-step adjusted; SIDAK SD, Sidak step-down adjusted; FDR BH, Benjamini & Hochberg (1995) step-up FDR control), and FDR BY (Benjamini & Yekutieli (2001) step-up FDR control).
